# IPD3 and IPD3L Function Redundantly in Rhizobial and Mycorrhizal Symbioses

**DOI:** 10.3389/fpls.2018.00267

**Published:** 2018-03-16

**Authors:** Yue Jin, Zixuan Chen, Jun Yang, Kirankumar S. Mysore, Jiangqi Wen, Jirong Huang, Nan Yu, Ertao Wang

**Affiliations:** ^1^College of Life and Environment Sciences, Shanghai Normal University, Shanghai, China; ^2^National Key Laboratory of Plant Molecular Genetics, CAS Center for Excellence in Molecular Plant Sciences, Institute of Plant Physiology and Ecology, Chinese Academy of Sciences, Shanghai, China; ^3^Collaborative Innovation Center of Crop Stress Biology, Institute of Plant Stress Biology, Henan University, Kaifeng, China; ^4^Plant Biology Division, Samuel Roberts Noble Foundation, Ardmore, OK, United States

**Keywords:** IPD3, IPD3L, nodule morphogenesis, phosphorylation, *Medicago truncatula*

## Abstract

Legume plants form symbiotic associations with either nitrogen-fixing bacteria or arbuscular mycorrhizal (AM) fungi, which are regulated by a set of common symbiotic signaling pathway genes. Central to the signaling pathway is the activation of the DMI3/IPD3 protein complex by Ca^2+^ oscillations, and the initiation of nodule organogenesis and mycorrhizal symbiosis. DMI3 is essential for rhizobial infection and nodule organogenesis; however, *ipd3* mutants have been shown to be impaired only in infection thread formation but not in root nodule organogenesis in *Medicago truncatula*. We identified an *IPD3*-like (*IPD3L*) gene in the *M. truncatula* genome. A single *ipd3l* mutant exhibits a normal root nodule phenotype. The *ipd3l/ipd3-2* double mutant is completely unable to initiate infection threads and nodule primordia. *IPD3L* can functionally replace *IPD3* when expressed under the control of the IPD3 promoter, indicating functional redundancy between these two transcriptional regulators. We constructed a version of IPD3 that was phosphomimetic with respect to two conserved serine residues (IPD3-2D). This was sufficient to trigger root nodule organogenesis, but the increased multisite phosphorylation of IPD3 (IPD3-8D) led to low transcriptional activity, suggesting that the phosphorylation levels of IPD3 fine-tune its transcriptional activity in the root nodule symbiosis. Intriguingly, the phosphomimetic version of IPD3 triggers spontaneous root-like nodules on the roots of *dmi3-1* and *dmi2-1* (*DMI2* is an LRR-containing receptor-like kinase gene which is required for Ca^2+^ spiking), but not on the roots of wild-type or *ipd3l ipd3-2* plants. In addition, fully developed arbuscules were formed in the *ipd3l ipd3-2* mutants but not the *ccamk/dmi3-1* mutants. Collectively, our data indicate that, in addition to *IPD3* and *IPD3L*, another new genetic component or other new phosphorylation sites of IPD3 function downstream of *DMI3* in rhizobial and mycorrhizal symbioses.

## Introduction

The symbiosis between the majority of land plants and arbuscular mycorrhizal (AM) fungi results in highly branched intracellular symbiotic structures called arbuscules, which can deliver soil phosphate to the host plant (Parniske, 2008; Oldroyd, 2013; Wang et al., 2017). In turn, the plant supplies AM fungi with lipid as a carbon source (Jiang et al., 2017; Luginbuehl et al., 2017). Legume plants also form symbiosis with soil rhizobia and develop a new root-derived organ called nodule in which differentiated bacteria convert atmospheric nitrogen into a form that can be assimilated by the host plant (Oldroyd, 2013). The establishment of both root nodule and mycorrhizal symbioses is initiated through recognizing lipochitooligosaccharides, namely nodulation (Nod) factors or mycorrhizal (Myc) factors by the LysM receptor kinase proteins, and is regulated by a set of common symbiotic signaling pathway genes (Limpens et al., 2003; Madsen et al., 2003; Radutoiu et al., 2003; Arrighi et al., 2006; Zhang et al., 2007, 2015).

Ca^2+^ oscillations are the earliest molecular responses to the Nod or Myc factors, and the DMI3-IPD3 (named CCaMK-CYCLOPS in *Lotus japonicus*) complex is required for the decoding of the calcium signal and initiation of nodule organogenesis in *Medicago truncatula* (Yano et al., 2008; Singh et al., 2014). CCaMK/DMI3 is a calcium- and calmodulin-dependent protein kinase (Levy et al., 2004; Mitra et al., 2004; Tirichine et al., 2006), and is composed of a CaM-binding site, a kinase domain, and 3 EF-hand motifs, and its catalytic activity is activated by either free or CaM-bound Ca^2+^ ions, suggesting that CCaMK/DMI3 might convert the Ca^2+^ signal into a protein phosphorylation read-out (Gleason et al., 2006; Tirichine et al., 2006). *ccamk-13*/*dmi3-1* mutants are completely deficient in both the root nodule and mycorrhizal symbioses. A *dmi3-1* mutant exhibits root hair swelling in response to the Nod factor, but fails to initiate infection threads or cortical cell divisions for nodule formation (Levy et al., 2004; Mitra et al., 2004). Gain of function of DMI3, by the deletion of the C-terminal regulatory domain DMI3 1-311, leads to spontaneous nodule formation in the absence of rhizobia (Gleason et al., 2006; Tirichine et al., 2006). Additionally, intraradical hyphae (Int-hyphae) and arbuscules cannot be formed in the *dmi3-1* mutants (Levy et al., 2004; Kistner et al., 2005). CCaMK/DMI3 acts as a central regulator in the root nodule and mycorrhizal symbioses; however, the mechanism underlying differentially activating root nodule symbiosis and arbuscular mycorrhizal symbiosis-related signal pathways by CCaMK/DMI3 is largely unknown.

*IPD3/CYCLOPS* encodes a nuclear-localized transcription factor with a coiled-coil motif, and is a direct phosphorylation substrate of CCaMK/DMI3 (Messinese et al., 2007; Yano et al., 2008). *ipd3* mutants of *M. truncatula* can develop an infection pocket or short impaired infection thread, and nodule primordia during root nodule symbiosis (Yano et al., 2008; Horváth et al., 2011; Ovchinnikova et al., 2011). The *Mtipd3* mutants can also form fully developed arbuscules in *M. truncatula* (Horváth et al., 2011). *CYCLOPS* is the homologous gene of *IPD3* in *L. japonicus. cyclops-3, Oscyclops* mutant alleles only develop Int-hypae during AM symbiosis in *L. japonicus* and rice, respectively (Chen et al., 2008; Yano et al., 2008; Horváth et al., 2011; Pimprikar et al., 2016). As a DNA-binding transcriptional activator, two phosphorylated serine residues (S50, S154) within the N-terminal negative regulatory domain of CYCLOPS are necessary for its activity in *L. japonicus*. A phosphomimetic version of CYCLOPS, in which two phosphorylated serine residues (S50, S154) are replaced by asparagic acid, can transactivate the expression of the nodulation-specific gene *NODULE INCEPTION* (*NIN*) and this is sufficient to trigger root nodule organogenesis in the absence of rhizobia (Soyano et al., 2014). However, the phosphomimetic version of CYCLOPS could not correct the plant's defective interaction with mycorrhizal fungi, indicating that other phosphorylation sites of *IPD3/CYCLOPS* were involved in AM symbiosis, or another unknown protein, exist downstream of DMI3 to control AM symbiosis in parallel with IPD3.

Recently, we found that IPD3 may function in a large protein complex containing other transcription factors such as DELLAs, Nodulation Signaling Pathway 2 (NSP2), and NSP1 to activate the expression of downstream genes in *M. truncatula* (Jin et al., 2016). DELLAs are the key negative regulators of gibberellin signaling (de Lucas et al., 2008; Feng et al., 2008) and rhizobia colonization is impaired in *della* mutants in *M. truncatula* (Fonouni-Farde et al., 2016; Jin et al., 2016). DELLAs and NSP1/NSP2 are GRAS (GAI, RGA, SCR)-type transcription factors involved in the root nodule symbiosis. Both *nsp1* and *nsp2* mutants cannot form infection pockets or initiate cortical cell division, and these mutants are therefore phenocopies of *dmi3-1* in *M. truncatula* (Kalo et al., 2005; Smit et al., 2005). *Mtipd3* mutants can develop nodule bumps and have impaired rhizobial infection in *M. truncatula* (Yano et al., 2008; Jin et al., 2016). This phenotype suggests genetic redundancy downstream of DMI3.

Here, we describe the identification and characterization of an *IPD3*-homologous gene, *IPD3-Like* (*IPD3L*) in *M. truncatula*. We show that the *ipd3l ipd3-2* double mutant is impaired in both rhizobial infection and root nodule organogenesis. We further show that phosphorylation levels of IPD3 fine-tune its transcriptional activity in the root nodule symbiosis. Our data also provide evidence that, in addition to *IPD3*/*IPD3L*, another new genetic component or other new phosphorylation sites of IPD3 function downstream of *DMI3* to control nodule morphogenesis and arbuscule formation.

## Materials and methods

### Phylogenetic analysis

We searched through BLAST in the phytozome database (http://www.phytozome.net/, Goodstein et al., 2012) for sequences similar to IPD3, and the results were inspected manually. Sequences were aligned with MUSCLE, followed by manual alignment.

Phylogenetic trees were constructed using an alignment of all length protein sequences with the neighbor-joining method with the option of pairwise deletion. To test inferred phylogeny, we used bootstraps with 1,000 bootstrap replicates (Tamura et al., 2011).

### Plant materials, bacterial and fungal strains and growth conditions

We obtained the *ipd3l* mutant (NF14178) from a *M. truncatula* mutant population generated at the Samuel Roberts Noble Foundation (http://medicago-mutant.noble.org/mutant/database.php). The insertion of a *Tnt1* retrotransposon in mutants was confirmed by PCR (for primers see Table S1). The double mutant *ipd3l ipd3-2* was generated by manual cross with *ipd3-2* (NF5939) as the female parent and *ipd3l* (NF14178) as the male parent. The progeny of the crosses was screened through PCR using primers shown in Table S1. Phenotypic analysis was subsequently performed on the homozygous mutant and the corresponding wild-type line (R108 or Jemalong A17).

Plant seeds were scarified with 98% sulfuric acid and plated onto 1% agar medium at 4°C in dark. After about 3 days, they were moved to 22°C overnight for germination. Then the seedlings were moved to a mixed soil containing 1:1 ratio of sand and perlite. For root nodule symbiosis, plants inoculated with *Sinorhizobium meliloti* 1021 (*Sm*1021) and were grown in a greenhouse at 22°C with a 16/8 h light/dark cycle at 22°C for 4 weeks. *S. meliloti* 1021 was incubated in liquid Luria-Bertani medium overnight with 400 mg/mL streptomycin selection. Bacteria were pelleted at 2,000 g for 15 min and resuspended in H_2_O to OD_600_ = 0.03.

For AM symbiosis, plants were coincubated with *Rhizophagus irregularis* for 2 months. To visualize the fungal structures, roots were stained in 0.2 mg/mL WGA (wheat germ agglutinin) Alexa-fluor 488 (Javot et al., 2007). Colonization levels specified as percent root length colonized were assessed by the modified gridline intersect method (McGonigle et al., 1990). *Agrobacterium rhizogenes*-mediated root transformations were done according to Boisson-Dernier and associates using strain ARqual (Boisson-Dernier et al., 2001).

### DNA constructs

For yeast two-hybrid constructs, the appropriate genes were cloned into the Gateway donor vector pEntry-topo-SD (Table S1), sequenced and then recombined into the pGBKT7GW and pGADT7GW vectors by LR reactions (Invitrogen). For subcellular localization vectors, *IPD3* and *IPD3L* were cloned into pSAT4-eGFP-N1 with appropriate restriction enzyme cleavage sites (Table S1). For protein expression in *Escherichia coli*, pET28a, and pMal-C2X were used (for primers see Table S1). For transient expression assays, the promoter of *ERN1* was fused to the pCAMBIA2300 vector. For transgenic hairy root vectors, *3XHA-IPD3*, and its point mutations (S14, S43, S50, T59, S81, S88, S155, and S407) were amplified and inserted into the Gateway donor vector pEntry-topo-SD and then recombined into the pK7GW2-R vectors by LR reactions. For GUS staining assay, the inferred *IPD3* promoter (1048 bp upstream of the start codon according to Ovchinnikova et al., 2011), and *IPD3L* promoter (3,000 bp upstream of the start codon) were cloned to the vector pEntry-topo-SD, and then recombined into pBGWFS7. For complementary experiments, full-length *IPD3L* CDS was amplified from cDNA, cloned to the vector pEntry-topo-SD, and then recombined into pK7WG2-R of which the p35S promoter was replaced by IPD3 promoter with appropriate restriction enzyme cleavage sites. All primers are listed in Table S1.

### Protein subcellular localization and yeast two-hybrid analysis

Plasmids were extracted according to the manual of NucleoBond Xtra Midi Plus (Macherey-Nagel). Isolation of *Arabidopsis* mesophyll protoplasts and transient gene expression were performed as previously described (Yoo et al., 2007). Enhanced green fluorescent protein (eGFP) fluorescence was recorded by a confocal laser scanning microscope (FluoView FV1000; Olympus).

Yeast two-hybrid analysis was carried out as described in the yeast handbook PT3024-1 (Clontech). The transformed AH109 yeast strains were selected on synthetic dropout (SD) plates lacking Leu and Trp (-LW). The expression of *HIS3* and *ADE2* reporter genes was assessed by colony growth of yeast strains transformed with DMI3, *IPD3* or *IPD3L* genes on SD-LWHA (-Leu, -Trp, -His, and -Ade) plates.

### GUS staining and enzymatic assays

Transgenic plants hairy roots which were mediated by *Agrobacterium rhizogenes*, containing promoter-GUS constructs were used for the GUS staining assay. The roots were putted into the staining buffer and a vacuum was applied for 10 min. Roots were then incubated for 12 h or 24 h at 37°C. For enzymatic GUS assays, leaf tissues were ground in liquid nitrogen and homogenized in the GUS extraction buffer (50 mM sodium phosphate, pH 7.5, 10 mM β-mercaptoethanol, 10 mM Na_2_EDTA, 0.1% Triton X-100, and 0.1% sodium lauryl-sarcosine) for total protein extraction. GUS activities were measured fluorimetrically using 1 mg of total protein extract as described previously (Boisson-Dernier et al., 2005).

### *In vitro* phosphorylation and mass spectrometric analysis

IPD3 was expressed from pET28a in *E. coli* strain Rosetta (TransGen Biotech). Expression products were affinity purified via nickel-agarose (Qiagen) under denaturing conditions using 8 M urea. Denatured proteins were refolded by stepwise dialysis (Yano et al., 2008). Purification of MBP-DMI3, HIS-IPD3, and HIS-IPD3L was performed according to the protocols of E8200 (New England Biolab) and of BioSprint96 (Qiagen). IPD3 was phosphorylated *in vitro* by DMI3 as described (Jin et al., 2016). Each reaction was carried out with 1 mg MBP-DMI3 protein, 0.2 mM CaCl_2_, 0.5 mM bovine CaM, 2 mg 6XHIS-IPD3 as substrate. Mass spectrometric analysis, in-gel digestion of phosphorylated IPD3, liquid chromatography (LC)-MS data acquisition and database searches were performed by Shanghai Applied Protein Technology Co. Ltd (http://www.aptbiotech.com).

### Gene expression analysis

Total RNA was extracted from *M. truncatula* hairy roots 4 weeks post transformation using the TransZol UP reagent (TransGen). RNA samples were treated with Ambion DNA Removal Kit and absence of DNA contamination was confirmed by PCR. First strand cDNA was synthesized with oligo (dT) 18 from 1 mg RNA with M-MLV reverse transcriptase (Takara). Real-time RT-PCR analysis was carried out in a CFX96 Real-Time PCR machine (BioRad) using 1 μl of diluted (1:20) cDNA in a total reaction volume of 10 μl containing SYBR Green Master Mix (Takara) and primers. Thermal cycling conditions were: 95°C 1 min, 45 cycles of 95°C 10 s, 60°C 30 s, followed by dissociation curve analysis. Relative expression was normalized to the reference gene ubiquitin. Mean and standard error values were calculated from three biological replicates. More than ten plants were used for RNA extraction.

### Transient expression in *Nicotiana benthamiana* leaves

*Agrobacterium tumefaciens* GV3101 strains containing binary vectors harboring either 3XHA-tagged IPD3 or its mutations and pro*ERN1:GUS* fusion were grown on LB medium supplemented with the appropriate antibiotics at 28°C, harvested and resuspended in suspension buffer (10 mM MgCl_2_, 10 mM MES, 200 μM acetosyringone, pH = 7.0) before infiltration of *Nicotiana benthamiana* leaves. Leaf discs were harvested 36 h post-infiltration and frozen in liquid nitrogen prior to quantitative enzymatic GUS fluorimetric assays. The expressed proteins in *Nicotiana benthamiana* leaves were detected by western blot analysis using anti-HA (Sigma) antibody (the uncropped versions of blots in Figure S7).

## Results

### Identification and cloning of an *IPD3*-like gene

In *ipd3-2* mutants, the development of infection threads is aborted and nodule development is prematurely arrested (Yano et al., 2008; Horváth et al., 2011; Ovchinnikova et al., 2011). The phenotype of *ipd3-2* is clearly different from *dmi3-1* mutants, in which both infection threads and nodule initiation are aborted. Therefore, we speculated that an *IPD3* homologous gene might function redundantly with IPD3 in the root nodule symbiosis. We searched for a homologous protein sequence in the phytozome database (https://phytozome.jgi.doe.gov/pz/portal.html) using the IPD3 full-length protein sequence, and a protein composed of 626 AA was found, which we named IPD3-like (IPD3L). We employed reverse-transcription polymerase chain reaction (RT-PCR) to amplify *IPD3L* cDNA, and found that there were 9 more nucleotides at the 256 site in the amplified fragment than predicted from the genome sequence (http://www.medicagogenome.org) (Data set S1). IPD3L has a longer C-terminus than IPD3 (Figure S2). A phylogenetic tree showed that IPD3L is more closely related to IPD3 than to an IPD3 homolog in the non-mycorrhizal plant *Arabidopsis*, suggesting that IPD3L might function in root nodule and mycorrhizal symbioses (Figure S1).

### The *ipd3l ipd3-2* double mutant is impaired in both rhizobial infection and root nodule development

IPD3 and DMI3 both localize to the nucleus and decode the calcium oscillation in the nucleus (Messinese et al., 2007; Yano et al., 2008). To test whether IPD3L also was localized in the nucleus, we constructed a vector expressing *IPD3L-eGFP* driven by a 35S promoter. *Arabidopsis* protoplasts expressing the control *p35S:eGFP* vector exhibited strong fluorescence in both cytoplasmic and nuclear compartments (Figure S3A). Fusion of eGFP to the full-length IPD3 and IPD3L resulted in specific accumulation of GFP signals in the nucleus (Figure S3A). Meanwhile, we found that IPD3L can interact with DMI3 in a yeast two-hybrid assay (Figure S3B). These results suggested that IPD3L might function redundantly with IPD3 in the root nodule and mycorrhizal symbioses.

**Figure 1:**
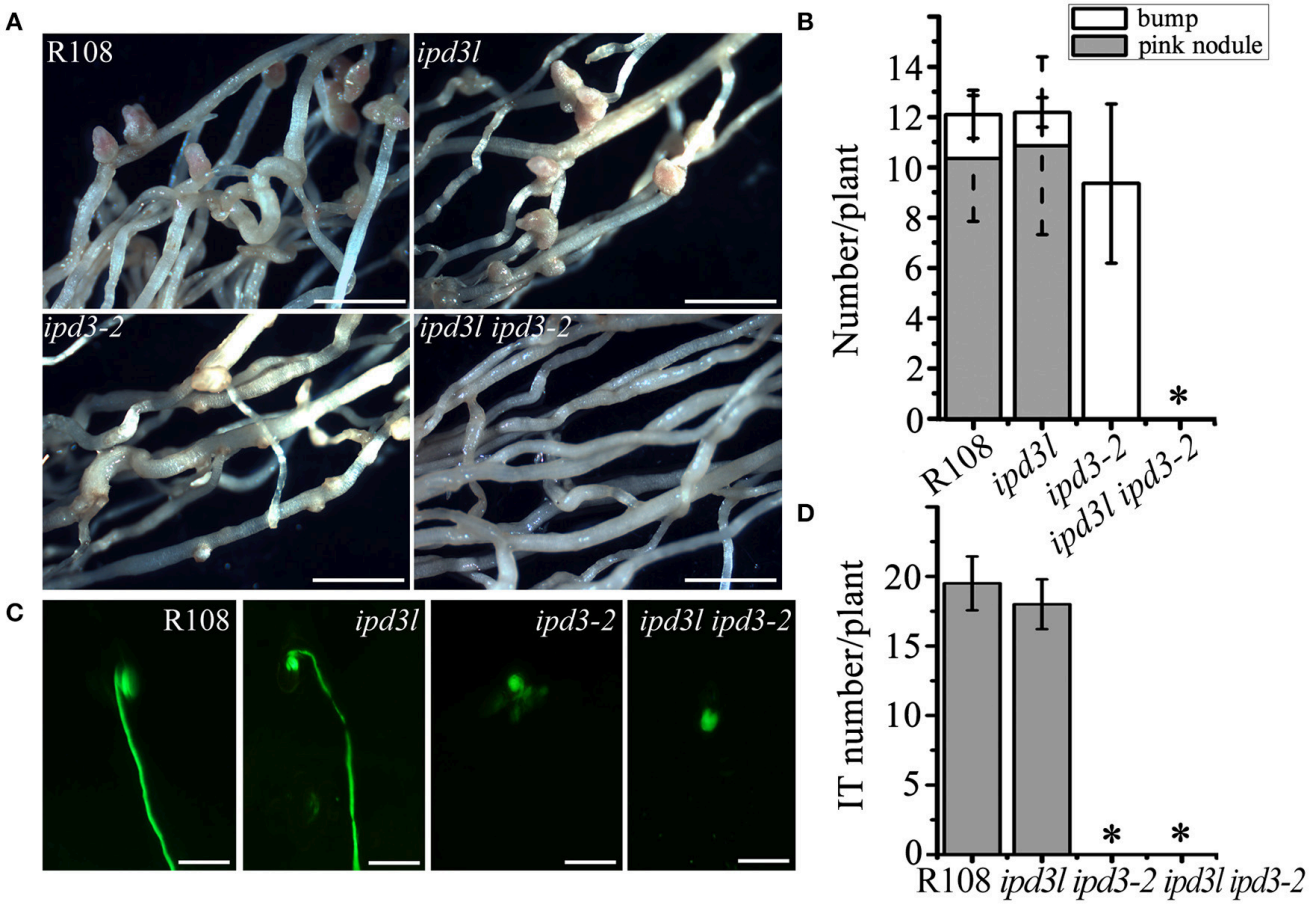
*ipd3l ipd3-2* mutant is impaired in nodule development. **(A)** Phenotypic analyses of wild-type R108, *ipd3l, ipd3-2*, and *ipd3l ipd3-2* double mutants at 4 weeks post-inoculation (wpi) with *Sinorhizobium meliloti* (strain 1021). Pink nodules were developed on R108 and *ipd3l* roots. Uninfected nodules (bump) were found on *ipd3-2* roots. No pink nodules or bumps were observed on the *ipd3l ipd3-2* double mutant roots. **(B)** Quantitative analysis of nodules grown on R108, *ipd3l, ipd3-2*, and *ipd3l ipd3-2* roots. **(C)** Representative images of infection threads in the wild-type, *ipd3l, ipd3-2* and *ipd3l ipd3-2* mutants. *S. meliloti* 1021 is labeled by green fluorescent protein (GFP). **(D)** Quantitative analysis of infection threads (IT) grown on R108, *ipd3l, ipd3-2*, and *ipd3l ipd3-2* roots. Scale bars correspond to 1 mm in **(A,B)** 100 μm in **(C)**. These experiments were repeated three times with similar results. The error bars indicate standard error. The asterisk indicates a significant decrease relative to the control with Student's *t*-test (^*^P ≤ 0.01).

To further explore the function of *IPD3L*, we ordered *ipd3l* mutants containing *Tnt1* insertions (NF14178) from a mutant population generated at the Samuel Roberts Noble Foundation (http://medicago-mutant.noble.org/mutant/database.php). Transcripts of *IPD3L* was absent in the *ipd3l* mutant line (Figures S2, S3). The rhizobial infection and nodule development in the *ipd3l* mutant were comparable to wild-type (R108) (Figure 1A). Considering the possibility of functional redundancy, we generated an *ipd3l ipd3-2* double mutant by crossing the *ipd3-2* (NF5939) with *ipd3l* (NF14178) mutant lines. Double mutants were obtained from the progeny through PCR analysis for further phenotypic analysis (Table S1). Intriguingly, no nodule primordia were formed in the *ipd3l ipd3-2* double mutants (Figures 1A,B), which is a phenocopy of the *dmi3-1* mutant. Further investigation showed that only infection pockets were formed on the roots of the *ipd3l ipd3-2* mutant (Figures 1C,D), indicating that *IPD3L* and *IPD3* function redundantly in the root nodule symbiosis.

### The expression of *IPD3L* in root nodule symbiosis

**Figure 2:**
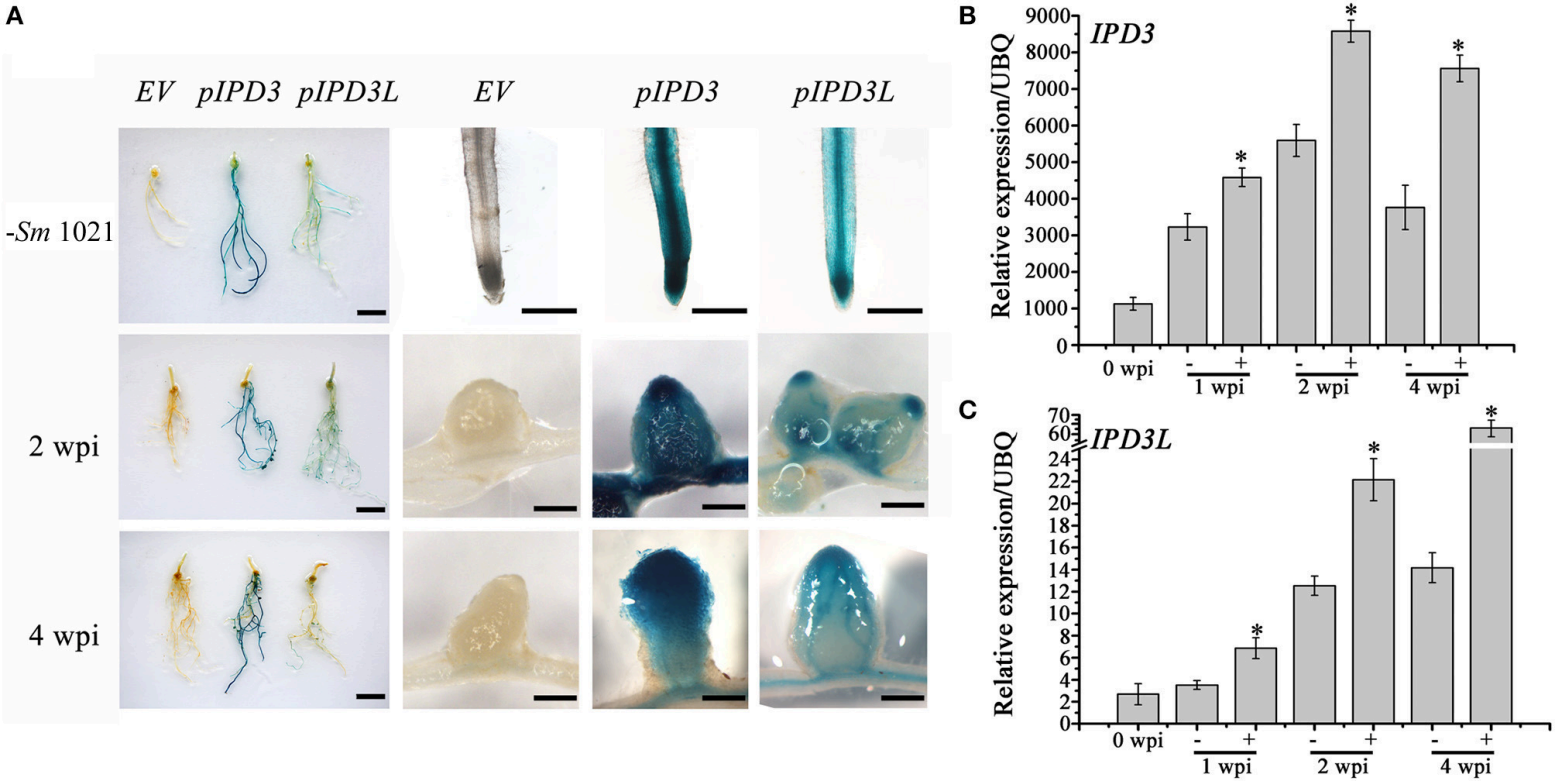
Expression pattern of *IPD3* and *IPD3L*. **(A)** GUS activity of the *pIPD3:GUS* and *pIPD3L:GUS* transformed plants at different time points with or without inoculation by *S. meliloti* 1021. The left panel shows the whole root system. These experiments were repeated more than three times. **(B)** Expression analyses of *IPD3* by real-time PCR at 1, 2, 4 wpi. **(C)** Expression analyses of *IPD3L* by real-time PCR at 1, 2, 4 wpi. Scale bars in the left panel correspond to 5 cm; other scale bars correspond to 100 μm. These experiments were repeated three times. More than 10 plants were used for RNA extraction. The error bars indicate standard error (*n* = 3). The asterisk indicates a significant decrease relative to the control (−*Sm*1021) with Student's *t*-test (^*^P ≤ 0.01).

To further investigate the role of *IPD3L* in the root nodule symbiosis, we compared the expression profiles of *IPD3* (*pIPD3:GUS*) and *IPD3L* (*pIPD3L:GUS*). *IPD3* and *IPD3L* expression in roots was detected in the absence of rhizobial inoculation (Figure 2A). In the rhizobia-inoculated roots, *IPD3* expression was detected strongly through the whole nodule, while *pIPD3L:GUS* expression was slightly detected in the meristem region and vascular tissues at 2 weeks post-inoculation (wpi) (Figure 2A). At 4 wpi, the *GUS* activity of IPD3 was detected most strongly in the region from the nodule meristem region to the nitrogen-fixation zone, and the expression pattern of *IPD3L* was very similar to that of *IPD3*, but *IPD3L* was expressed at much lower levels at 2 and 4 wpi (Figure 2A). To verify the difference in the expression levels, real-time-PCR was further employed to reveal the expression levels of *IPD3* and *IPD3L. IPD3* expression levels were approximately 100 times higher than that of *IPD3L* in roots with or without rhizobial inoculation (Figures 2B,C). Thus, our results showed that *IPD3L* functions redundantly with *IPD3*, although the expression level of *IPD3L* is much lower than *IPD3* in roots.

### *IPD3L* can functionally replace *IPD3* when expressed under the control of the *IPD3* promoter

**Figure 3:**
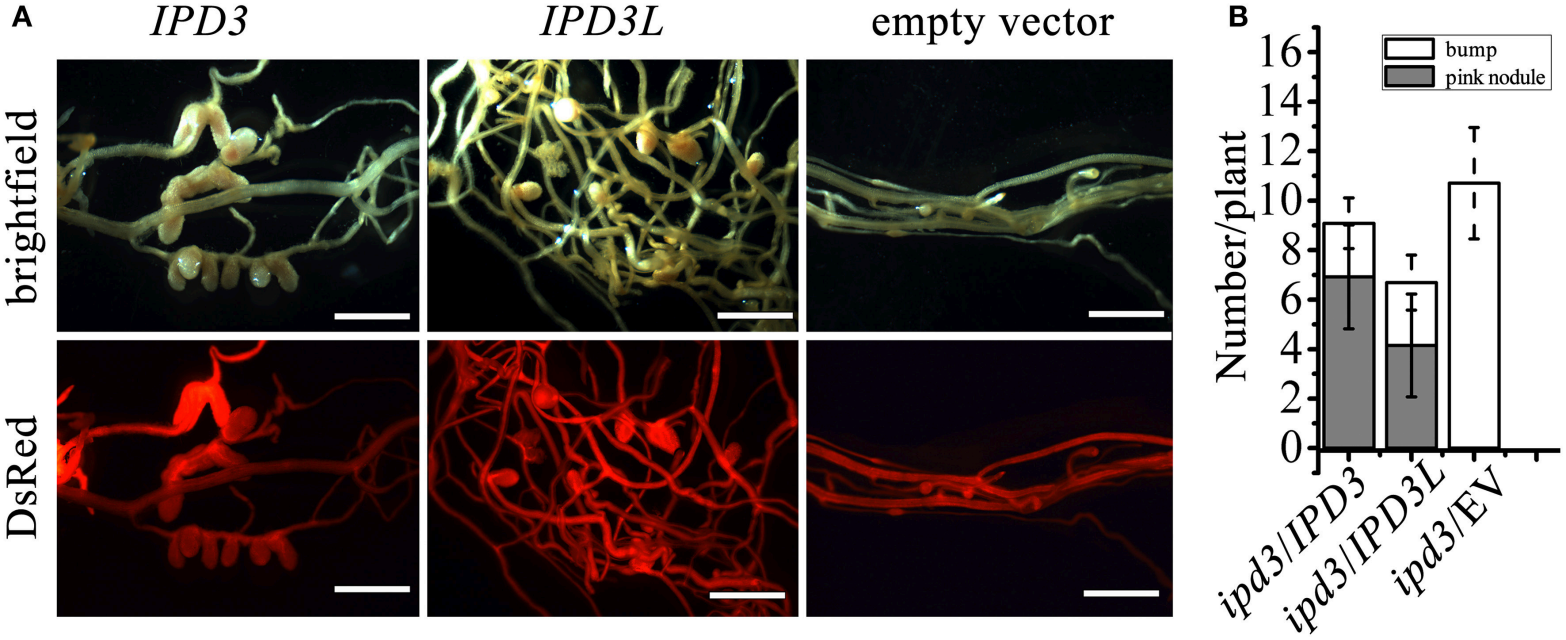
*IPD3L* rescues the phenotype of *ipd3-2*. **(A)** The nodulation phenotypes of *ipd3-2* mutant roots transformed with *IPD3*, or *IPD3L* or the empty vector control (expressed under the control of the 1.1 kb *IPD3* promoter) at 4 wpi. Transformed roots were screened with DsRed reporter (contained in the binary vector). **(B)** Quantitative analysis of nodule number on *ipd3-2* mutant roots transformed with *IPD3*, or *IPD3L* or the empty vector control at 4 wpi. Scale bars correspond to 1 mm. These experiments were repeated three times with similar results.

Only short infection threads and bumps developed on the *ipd3-2* mutant roots, whereas the normal pink nodules were observed on the *ipd3l* mutant roots (Figure 1). This implies that *IPD3L* and *IPD3* also function differently in the root nodule symbiosis. To address the question of whether *IPD3L* can functionally replace *IPD3*, cross-complementation studies were performed by transforming an *ipd3-2* mutant line with *IPD3L* driven by the *IPD3* promoter. Fully developed and infected nodules were formed on *ipd3-2* hairy roots transformed with *IPD3L*, but not on *ipd3-2* hairy roots transformed with the empty vector (Figure 3). Thus, when expressed under the control of the *IPD3* promoter, *IPD3L* can functionally replace *IPD3* in the root nodule symbiosis.

### Serine residue phosphorylation of IPD3 fine-tunes root nodule symbiosis

The availability of the non-nodulating double mutant *ipd3l ipd3-2* opened the possibility to characterize different phosphoablative/-mimetic IPD3 variants. No pink nodules were observed on *ccamk-3* roots transformed with *CYCLOPS-S50D-S154D* in *L. japonicus* (Singh et al., 2014). To identify other amino acids of IPD3 phosphorylated by DMI3 functioned in the root nodule symbiosis, we purified 6 × HIS-IPD3 protein and MBP-DMI3 protein from *E. coli* and performed DMI3-mediated IPD3 phosphorylation experiments *in vitro*. Three phosphorylated serine residues (S14, S81, S88) and one phosphorylated threonine residue (T59) were detected by mass spectrometry (Figure S5A). Among these sites, only one residue (S14) had a corresponding phosphorylation site in CYCLOPS (Singh et al., 2014). This discrepancy may be due to the different Ca^2+^ concentrations used in the studies. All together, eight potential phosphorylated residues of IPD3 protein (S14, S43, S50, T59, S81, S88, S155, and S407), including the S43 reported by Grimsrud et al. (2010), have been found so far from alignment between IPD3 and CYCLOPS (Figure S5B). This data suggest that DMI3 could phosphorylate additional residues in IPD3-2D and that these sites could be important for full IPD3 function.

To examine whether phosphorylation of the newly identified sites is essential for the root nodule symbiosis, we generated single phosphoablative mutant versions of IPD3 and analyzed the restoration of the root nodule symbiosis in transformed *ipd3l ipd3-2* mutants with these phosphoablative mutant versions. In these variants, T59, S81, and S88 were individually substituted by alanine (A) (Table S2). The number and morphology of pink nodules on the hairy roots of plants containing alanine replacements at the three sites was the same as the control. Therefore, we further generated multisite phosphoablative/-mimetic mutant versions of S14, S43, S50, T59, S81, S88, S155, and S407 to test their function in nodule formation. Replacing serine/threonine with aspartate (D) can result in a gain-of-function activity of the protein (phosphomimetic version of the protein). We found that the IPD3-2D version (S50, S155) could rescue nodule formation on the *ipd3l ipd3-2* double mutant (Table S3). Strikingly, IPD3-8D decreased the nodule number on the *ipd3l ipd3-2* mutant even though the morphology of pink nodules is not different from that on the wild-type plants. To test whether this phenotype was caused by some instability of the protein, we performed a western blot with an anti-HA-HRP antibody. We found that 3xHA-IPD3-WT and 3xHA-IPD3-8D were expressed at similar levels in *ipd3l ipd3-2* mutant roots (Figure S6A).

**Figure 4:**
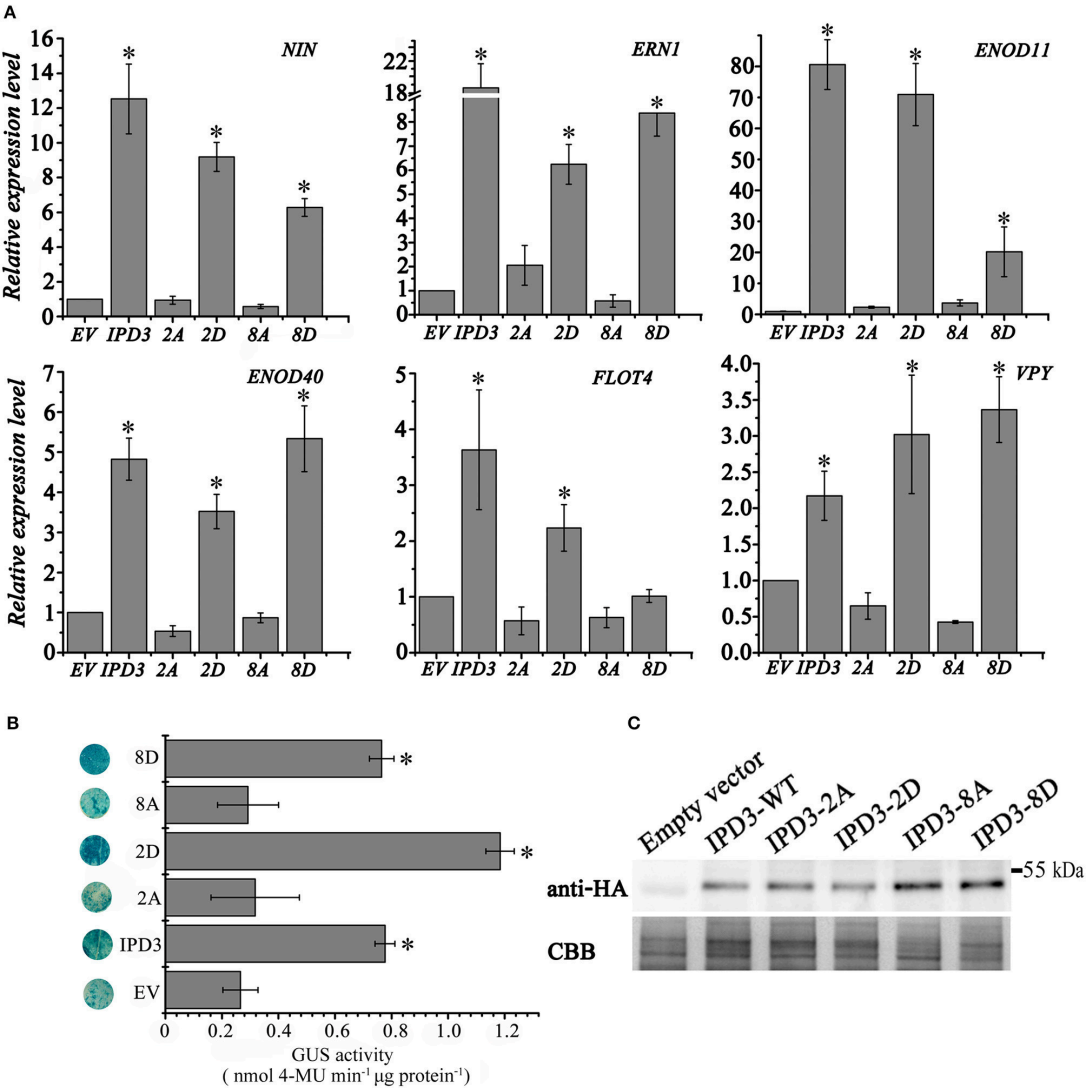
Root nodule symbiosis (RNS) related gene expression is promoted by IPD3 and phosphoablative/-mimetic IPD3 variants. **(A)** The relative expression levels of RNS-specific genes revealed by real-time PCR using the transgenic hairy roots of *M. truncatula* at 4 wpi. These experiments were repeated three biological times. More than ten plants were used for RNA extraction. **(B)** Transactivation assay of IPD3 in *Nicotiana benthamiana* leaves. 3xHA-IPD3-WT, 3xHA-IPD3-2A, 3xHA-IPD3-2D, 3xHA-IPD3-8A, or 3xHA-IPD3-8D was co-injected in *N. benthamiana* leaves with the *pERN1:GUS* reporter. GUS activity of leaf discs was determined histochemically and quantitatively as described previously (Boisson-Dernier et al., 2005). **(C)** Western blots revealed the protein content of 3xHA-IPD3-WT, 3xHA-IPD3-2A, 3xHA-IPD3-2D, 3xHA-IPD3-8A, 3xHA-IPD3-8D in *N. benthamiana* leaves. These experiments were repeated three times. The error bars indicate standard error (*n* = 3). The asterisk indicates a significant decrease relative to the control (EV) with Student's *t*-test (^*^P ≤ 0.01).

Consistent with the decreased nodule number phenotype, the expression level of nodule specific genes, namely *NIN, ERN1, Enod11* and *FLOT4* were lower in the *IPD3-8D* transformed hairy roots than that in *IPD3-WT* transgenic roots (Figure 4), but higher than that in the empty vector, *IPD3-2A*, and *IPD3-8A* roots. Meanwhile, we examined the transcriptional activity of different phosphomimetic versions of IPD3 in *N. benthamiana* leaves. Interestingly, empty vector, IPD3-2A, and IPD3-8A could cause a faint reporter gene expression, while IPD3 and IPD3-8D were sufficient to induce a significant expression of *ERN1*. IPD3-2D could further active expression of *ERN1* in *N. benthamiana* leaves (Figure S7). Together, our data suggest that IPD3-2D plays a positive role, while IPD3-8D plays a negative role in the root nodule symbiosis. However, the detail of the mechanism needs to be further explored.

CYCLOPS has been identified as a phosphorylation substrate of CCaMK and only uninfected nodules were observed on *ccamk-3* roots transformed with CYCLOPS-S50D-S154D in *L. japonicus* (Singh et al., 2014). Consistent with this, no pink nodules were found on *dmi3-1* roots transformed with IPD3-2D and IPD3-8D at 4 wpi in *M. truncatula* (Table S4), indicating that this multisite phosphorylation of IPD3 (IPD3-2D/8D) by DMI3 is not sufficient to trigger the downstream signaling for rhizobial infection.

### IPD3-2D induces spontaneous root nodules

**Figure 5:**
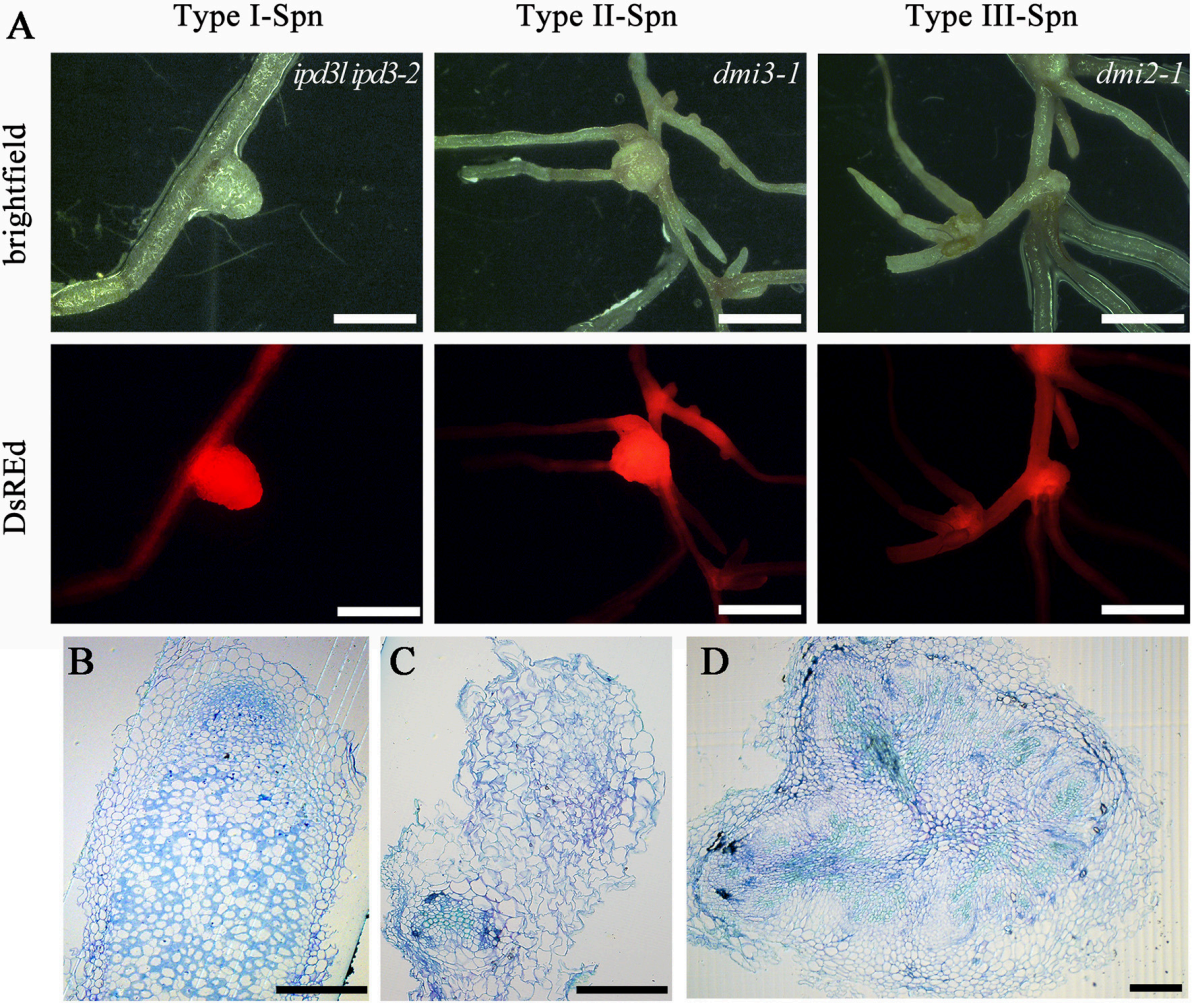
IPD3-2D triggers spontaneous root-like nodules. **(A)** Type I spontaneous nodules developed on the *ipd3l ipd3-2* hairy roots transformed with the phosphomimetic IPD3-2D mutant version, Type II and III developed on the hairy roots of *dmi3-1* and *dmi2-1* transformed with the phosphomimetic IPD3-2D mutant version. Spontaneous nodules were observed and scored at 8 weeks transformed into the soil. **(B)** Toluidine blue-stained section of a pink nodule induced by *S. meliloti* 1021. **(C)** Toluidine blue-stained sections of type I spontaneous root-like nodules. **(D)** Toluidine blue-stained sections of type II spontaneous root-like nodules. These experiments were repeated three times with similar results. Scale bars in **(A)** correspond to 1 mm; scale bars in **(B–D)** correspond to 0.1 mm.

Gain of function versions of CCaMK/DMI3 are sufficient to trigger spontaneous nodule organogenesis in the absence of rhizobia (Gleason et al., 2006; Tirichine et al., 2006). We found that IPD3-2D was also sufficient to trigger spontaneous nodules on the wild-type and *ipd3l ipd3-2* roots in the absence of rhizobia (Figure 5, Table S5), which is consistent with the data reported in *L. japonicus* (Singh et al., 2014). Strikingly, the spontaneous nodules on *dmi3-1* and *dmi2-1* roots triggered by IPD3-2D were root-like nodules with many similarities to those on *Mtnoot* (*nodule root* in *M. truncatula*) and *Pscoch* (*cochleata in Pisum sativum*) roots, which are characterized by the abnormal development of roots from the nodule (Couzigou et al., 2012) (Figure 5). This observation indicated that a signal was activated by CCaMK/DMI3 to maintain the normal spontaneous development of nodules triggered by IPD3-2D. This signal may be a new protein or some new phosphoralation sites of IPD3. In addition, the number of nodules on the hairy roots transformed with IPD3-8D vectors was significantly decreased compared with roots transformed with IPD3-2D in *ipd3l ipd3-2, dmi3-1*, and *dmi2-1* mutants (Table S5).

Since IPD3-2D induced spontaneous root nodules on *dmi3-1* mutants, we asked whether formation of spontanious nodules induced by an autoactive form of DMI3 (DMI3 1-311) is dependent on IPD3. We introduced the DMI3 1–311 into the *ipd3l ipd3-2* mutant via hairy root transformation. Only several surface protuberances (3/25) were found (Figure S6), but no spontaneous nodules were formed on *ipd3l ipd3-2* mutants, indicating that *IPD3/IPD3L* is required for spontaneous nodules induced by expression of a deregulated form of DMI3.

### Fully developed arbuscules are formed in *ipd3l ipd3-2* mutants

**Figure 6:**
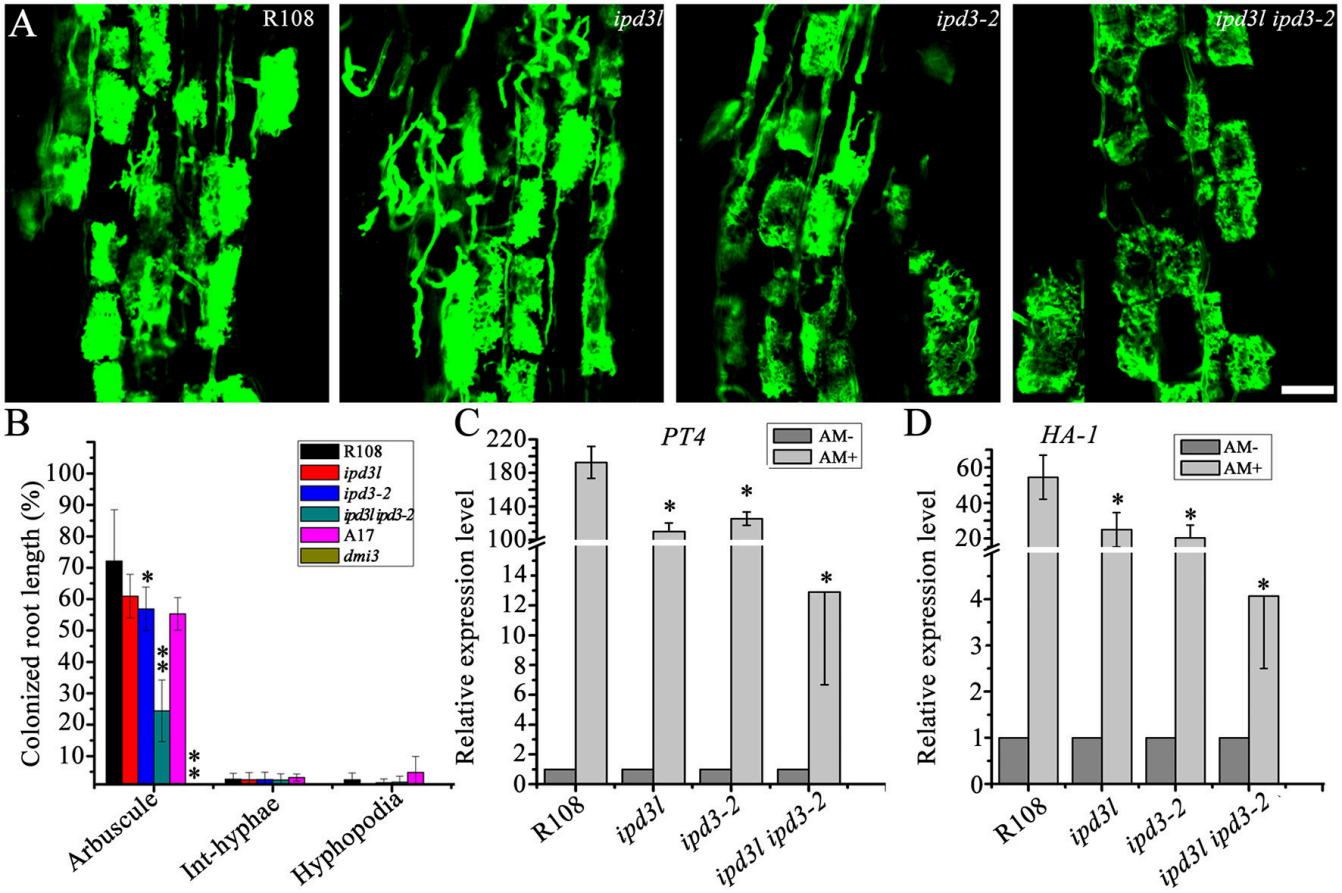
Fully developed arbuscules are formed in the *ipd3l ipd3-2* mutant roots. **(A)** Laser scanning confocal images of wild-type, *ipd3l, ipd3-2*, and *ipd3l ipd3-2* mutants at 8 wpi. The fungus was stained with 0.2 mg/mL WGA-Alexa-Fluor 488. Scale bars correspond to 20 μm. **(B)** Percent root length colonization of wild-type, *ipd3-2, ipd3l*, and *ipd3l ipd3-2* roots by *Rhizophagus irregularis* at 45 days post-inoculation (dpi). The degree of root colonization was determined by gridline intersect method. These experiments were repeated four times with similar results. The asterisk indicates a significant decrease compared to the control with Student's *t*-test (^*^P ≤ 0.05; ^**^P ≤ 0.01). **(C,D)** Expression analyses of arbuscular mycorrhizal specific marker genes by real-time PCR. These experiments were repeated three biological times. More than ten plants were used for RNA extraction. The error bars indicate standard error (*n* = 3). The asterisk indicates a significant decrease compared to the control with Student's *t*-test (^*^P ≤ 0.01).

It has been shown that *IPD3* plays an important role in mycorrhizal symbiosis (Yano et al., 2008; Horváth et al., 2011; Pimprikar et al., 2016). Therefore, we assessed whether *IPD3L* also functions redundantly in mycorrhizal symbiosis. Wild-type, *ipd3-2, ipd3l*, and *ipd3l ipd3-2* roots inoculated with sands mixed with *Rhizophagus irregularis*. Surprisingly, fully developed arbuscules were found in the *ipd3l ipd3-2* roots (Figure 6A), although the frequency of fungal colonization was significantly reduced in the *ipd3l ipd3-2* mutant compared with wild-type plants (Figures 6B,C). In *dmi3-1* mutants, mycorrhizal hyphae were observed only on the root surface. Interestingly, our results differ from previous observations of *ipd3-2* roots (Horváth et al., 2011), where fully developed arbuscules were not observed in the mutant roots inoculated with *R. intraradices*. To verify our results, we repeated this experiment four times under our conditions, and highly branching arbuscules were consistently observed in roots of both the *ipd3-2* and the *ipd3l ipd3-2* double mutant (Figure 6). Consistent with reduced AM colonization, the expression levels of AM marker genes, *PT4 (PHOSPHATE TRANSPORTER 4)* and *HA-1(H*^+^*-ATPase)* (Javot et al., 2007; Wang et al., 2014), were seriously decreased in the *ipd3l ipd3-2* plants. Taken together, our data indicate that another genetic component, in addition to *IPD3*/*IPD3L*, functions downstream of *DMI3* to regulate arbuscule development in mycorrhizal roots.

## Discussion

The establishment of nitrogen-fixing nodules in legumes involves in the decoder of calcium spiking and regulation network of transcription factors. Forward and reverse genetic approaches have demonstrated that these transcription factors play central roles in root nodule symbiosis. However, despite clear-cut infection thread defect phenotypes, many transcription factors mutants, such as *ern1, ipd3, nf-ya1*, are still able to partially initiate early symbiotic signaling to form nodule primordia (Middleton et al., 2007; Yano et al., 2008; Horváth et al., 2011; Ovchinnikova et al., 2011; Cerri et al., 2012, 2016; Laloum et al., 2014; Laporte et al., 2014). Recent research has shown gene redundancy in the role of *ERNs* and *NF-YAs* in the root nodule symbiosis (Cerri et al., 2012, 2016; Laloum et al., 2014; Laporte et al., 2014). Our research has shown that the *ipd3l ipd3-2* double mutant exhibits a very severe symbiotic defect phenotype, being unable to form infection threads and nodules (Figure 1) and *IPD3L* can functionally replace *IPD3* when expressed under the control of the *IPD3* promoter (Figure 3). Thus, we conclude that *IPD3L* is a potential transcription factor that functions redundantly with *IPD3* to regulate root nodule symbiosis. These findings also indicate that *IPD3* and *IPD3L* may possess equivalent biological activities when expressed at similar levels in identical tissues. However, the expression levels and/or tissue specificity of endogenous *IPD3L* are not sufficient and/or appropriate to functionally replace *IPD3*, when the *IPD3* gene is inactivated (Figures 1, 2). We conclude that the functional specialization of these symbiotic transcription factors might occur primarily via the evolution of promoter specificity rather than by the divergence of protein functions.

In this study, we identified phosphorylation sites (S14, S43, S50, T59, S81, S88, S155, and S407) on IPD3 and found that the *IPD3-2D* (S50, S155) could rescue the nodule formation on the *ipd3l ipd3-2* double mutant roots (Table S3). Strikingly, IPD3-8D, the phosphomimetic sites decreased the nodule number compared to IPD3-WT or IPD3-2D (Tables S3, S4) and the expression level of root nodule symbiotic genes was also decreased in the *IPD3-8D* transgenic hairy roots. We speculate that the multisite phosphorylation of IPD3 (*IPD3-8D*) might function as a code to positively or negatively regulate root nodule symbiosis.

**Figure 7:**
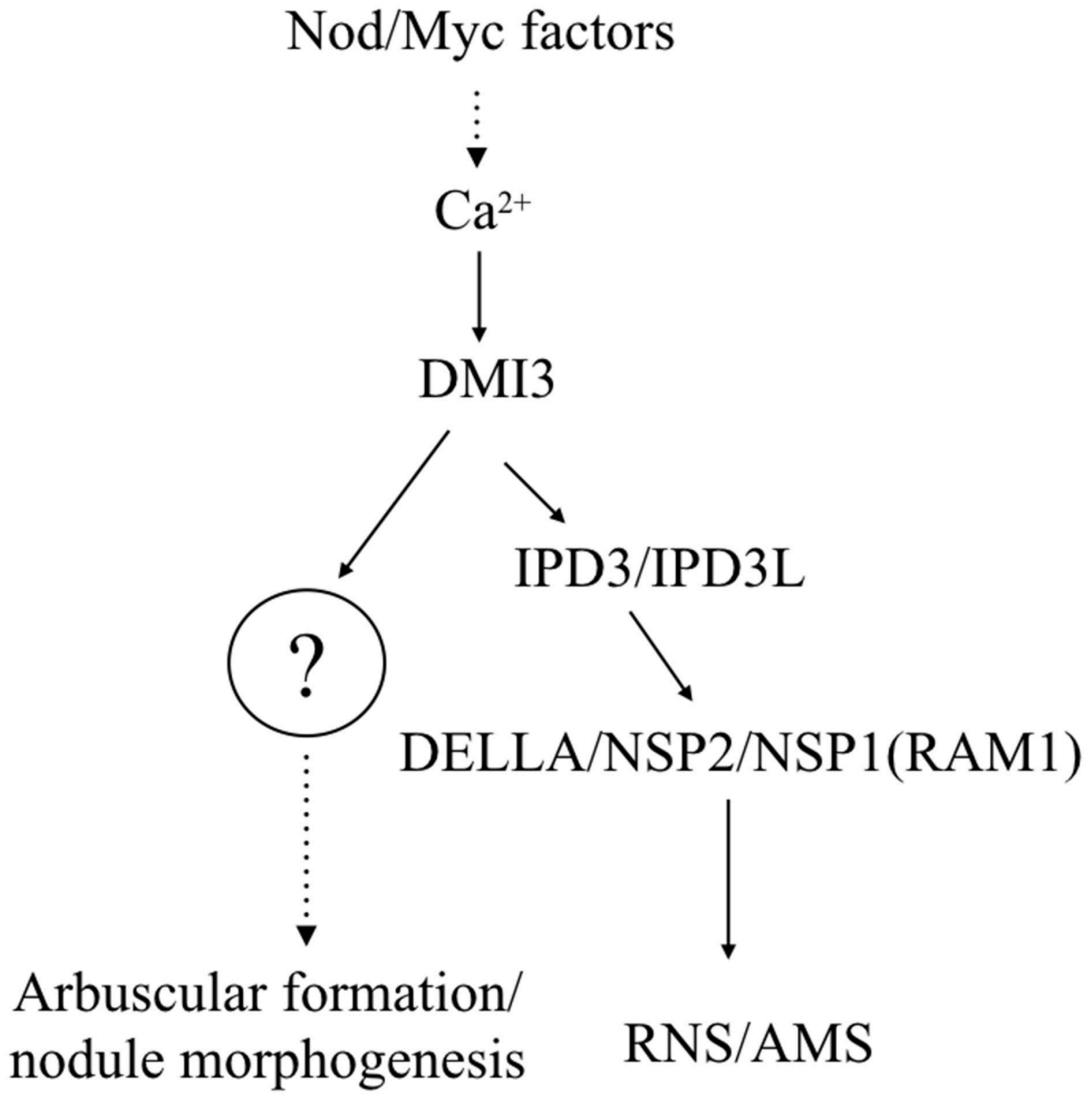
Working model of IPD3s transcription factors in rhizobial and mycorrhizal symbioses. These signaling pathways are an amalgamation of genetic analyses in *Lotus japonicus* and *Medicago truncatula*; gene names are indicated for *M. truncatula*. Calcium- and calmodulin-dependent protein kinase (CCaMK/DMI3) is responsible for decoding the calcium spiking and phosphorylates IPD3. In this model, the phosphorylation level of IPD3 controls the ability of IPD3 to bind the *ERN1* promoter. Interestingly, an unknown protein or other phosphralation sites of IPD3 may help to retain the stability of the IPD3 complex, or may act in parallel with IPD3 through binding DELLAs to activate downstream gene expression to initiate arbuscular formation and nodule morphogenesis.

Autoactive gain of function variants of CCaMK/DMI3 resulted in spontaneous nodule organogenesis in the absence of rhizobia (Gleason et al., 2006; Tirichine et al., 2006). CYCLOPS-2D can also trigger the spontaneous formation of root nodules in the absence of rhizobia (Singh et al., 2014). We revealed that IPD3-2D can promote spontaneous nodule formation on *ipd3l ipd3-2* hairy roots and also promote spontaneous root-like nodules on the *dmi2-1* and *dmi3-1* hairy roots (Figure 5 and Table S5). Furthermore, the spontaneous root-like nodules were not observed on the *dmi3-1* roots transformed with the autoactive DMI3 1-311 variant. Our current data indicates that an unknown protein and other phosphorylation sites of IPD3 activated by CCaMK/DMI3 are required to maintain nodule morphogenesis in the root nodule symbiosis (Figure 7).

Previous studies has shown that the *ipd3-2* roots showed the presence of intraradical and extraradical hyphae and pigmented cells, but no arbuscules inoculated with purified *Glomus intraradices* spores, while The *ipd3-1* plants displayed normal arbuscule development with lower frequency of colonization (Yano et al., 2008; Horváth et al., 2011; Pimprikar et al., 2016). Under our condition, fully developed arbuscules were also found in the *ipd3-2* and *ipd3l ipd3-2* roots inoculated with sands mixed with *R. intraradices* (Figure 6). This different phenotype of *ipd3-2* may be caused by the different AM fungi or the different growth conditions. We also propose that another genetic component, in addition to *IPD3/IPD3L*, functions downstream of *DMI3* to regulate arbuscule development during mycorrhizal symbiosis.

## Author contributions

YJ, EW, and NY conceived the original screening and research plans; YJ performed most of the experiments; YJ, EW, and NY conceived the project and wrote the article with contributions from all the authors.

### Conflict of interest statement

The authors declare that the research was conducted in the absence of any commercial or financial relationships that could be construed as a potential conflict of interest.

## References

[B1] Arrighi J. F. Barre A. Ben Amor B. Bersoult A. Soriano L. C. Mirabella R.. (2006). The *Medicago truncatula* lysin motif-receptor-like kinase gene family includes *NFP* and new nodule-expressed genes. Plant Physiol. 142, 265–279. 10.1104/pp.106.084657 16844829 PMC1557615

[B2] Boisson-Dernier A. Andriankaja A. Chabaud M. Niebel A. Journet E. P. Barker D. G.. (2005). *MtENOD11* gene activation during rhizobial infection and mycorrhizal arbuscule development requires a common AT-rich-containing regulatory sequence. Mol. Plant Microbe Interact. 18, 1269–1276. 10.1094/MPMI-18-1269 16478046

[B3] Boisson-Dernier A. Chabaud M. Garcia F. Becard G. Rosenberg C. Barker D. G. (2001). *Agrobacterium rhizogenes*-transformed roots of *Medicago truncatula* for the study of nitrogen-fixing and endomycorrhizal symbiotic associations. Mol. Plant Microbe Interact. 14, 695–700. 10.1094/MPMI.2001.14.6.695 11386364

[B4] Cerri M. R. Frances L. Kelner A. Fournier J. Middleton P. H. Auriac M. C.. (2016). The Symbiosis-related *ERN* transcription factors act in concert to coordinate rhizobial host root infection. Plant Physiol. 171, 1037–1054. 10.1104/pp.16.00230 27208242 PMC4902606

[B5] Cerri M. R. Frances L. Laloum T. Auriac M. C. Niebel A. Oldroyd G. E.. (2012). *Medicago truncatula* ERN transcription factors: regulatory interplay with NSP1/NSP2 GRAS factors and expression dynamics throughout rhizobial infection. Plant Physiol. 160, 2155–2172. 10.1104/pp.112.203190 23077241 PMC3510138

[B6] Chen C. Ane J. M. Zhu H. (2008). OsIPD3, an ortholog of the *Medicago truncatula* DMI3 interacting protein IPD3, is required for mycorrhizal symbiosis in rice. New Phytol. 180, 311–315. 10.1111/j.1469-8137.2008.02612.x 18761634

[B7] Couzigou J. M. Zhukov V. Mondy S. Abuel Heba G. Cosson V. Ellis T. H.. (2012). NODULE ROOT and COCHLEATA maintain nodule development and are legume orthologs of Arabidopsis BLADE-ON-PETIOLE genes. Plant Cell 24, 4498–4510. 10.1105/tpc.112.103747 23136374 PMC3531848

[B8] de Lucas M. Daviere J. M. Rodriguez-Falcon M. Pontin M. Iglesias-Pedraz J. M. Lorrain S.. (2008). A molecular framework for light and gibberellin control of cell elongation. Nature 451, 480–484. 10.1038/nature06520 18216857

[B9] Feng S. Martinez C. Gusmaroli G. Wang Y. Zhou J. Wang F.. (2008). Coordinated regulation of *Arabidopsis thaliana* development by light and gibberellins. Nature 451, 475–479. 10.1038/nature06448 18216856 PMC2562044

[B10] Fonouni-Farde C. Tan S. Baudin M. Brault M. Wen J. Mysore K. S.. (2016). DELLA- mediated gibberellin signalling regulates Nod factor signalling and rhizobial infection. Nat. Commun. 7:12636. 10.1038/ncomms12636 27586842 PMC5025792

[B11] Gleason C. Chaudhuri S. Yang T. Munoz A. Poovaiah B. W. Oldroyd G. E. (2006). Nodulation independent of rhizobia induced by a calcium-activated kinase lacking autoinhibition. Nature 441, 1149–1152. 10.1038/nature04812 16810256

[B12] Goodstein D. M. Shu S. Howson R. Neupane R. Hayes R. D. Fazo J.. (2012). Phytozome: a comparative platform for green plant genomics. Nucleic Acids Res. 40, D1178–D1186. 10.1093/nar/gkr944 22110026 PMC3245001

[B13] Grimsrud P. A. den Os D. Wenger C. D. Swaney D. L. Schwartz D. Sussman M. R.. (2010). Large-scale phosphoprotein analysis in *Medicago truncatula* roots provides insight into in vivo kinase activity in legumes. Plant Physiol. 152, 19–28. 10.1104/pp.109.149625 19923235 PMC2799343

[B14] Horváth B. Yeun L. H. Domonkos A. Halasz G. Gobbato E. Ayaydin F.. (2011). *Medicago truncatula IPD3* is a member of the common symbiotic signaling pathway required for rhizobial and mycorrhizal symbioses. Mol. Plant Microbe Interact. 24, 1345–1358. 10.1094/MPMI-01-11-0015 21692638

[B15] Javot H. Penmetsa R. V. Terzaghi N. Cook D. R. Harrison M. J. (2007). A *Medicago truncatula* phosphate transporter indispensable for the arbuscular mycorrhizal symbiosis. Proc. Natl. Acad. Sci. U.S.A. 104, 1720–1725. 10.1073/pnas.0608136104 17242358 PMC1785290

[B16] Jiang Y. Wang W. Xie Q. Liu N. Liu L. Wang D.. (2017). Plants transfer lipids to sustain colonization by mutualistic mycorrhizal and parasitic fungi. Science 356, 1172–1175. 10.1126/science.aam9970 28596307

[B17] Jin Y. Liu H. Luo D. Yu N. Dong W. Wang C.. (2016). DELLA proteins are common components of symbiotic rhizobial and mycorrhizal signalling pathways. Nat. Commun. 7:12433. 10.1038/ncomms12433 27514472 PMC4990646

[B18] Kalo P. Gleason C. Edwards A. Marsh J. Mitra R. M. Hirsch S.. (2005). Nodulation signaling in legumes requires NSP2, a member of the GRAS family of transcriptional regulators. Science 308, 1786–1789. 10.1126/science.1110951 15961668

[B19] Kistner C. Winzer T. Pitzschke A. Mulder L. Sato S. Kaneko T.. (2005). Seven Lotus japonicus genes required for transcriptional reprogramming of the root during fungal and bacterial symbiosis. Plant Cell 17, 2217–2229. 10.1105/tpc.105.032714 15980262 PMC1182484

[B20] Laloum T. Baudin M. Frances L. Lepage A. Billault-Penneteau B. Cerri M. R.. (2014). Two *CCAAT*-box-binding transcription factors redundantly regulate early steps of the legume-rhizobia endosymbiosis. Plant J. 79, 757–768. 10.1111/tpj.12587 24930743

[B21] Laporte P. Lepage A. Fournier J. Catrice O. Moreau S. Jardinaud M. F.. (2014). The *CCAAT* box-binding transcription factor *NF-YA1* controls rhizobial infection. J. Exp. Bot. 65, 481–494. 10.1093/jxb/ert392 24319255 PMC3904707

[B22] Levy J. Bres C. Geurts R. Chalhoub B. Kulikova O. Duc G.. (2004). A putative Ca^2+^ and calmodulin-dependent protein kinase required for bacterial and fungal symbioses. Science 303, 1361–1364. 10.1126/science.1093038 14963335

[B23] Limpens E. Franken C. Smit P. Willemse J. Bisseling T. Geurts R. (2003). LysM domain receptor kinases regulating rhizobial Nod factor-induced infection. Science 302, 630–633. 10.1126/science.1090074 12947035

[B24] Luginbuehl L. H. Menard G. N. Kurup S. Van Erp H. Radhakrishnan G. V. Breakspear A.. (2017). Fatty acids in arbuscular mycorrhizal fungi are synthesized by the host plant. Science 356, 1175–1178. 10.1126/science.aan0081 28596311

[B25] Madsen E. B. Madsen L. H. Radutoiu S. Olbryt M. Rakwalska M. Szczyglowski K.. (2003). A receptor kinase gene of the LysM type is involved in legume perception of rhizobial signals. Nature 425, 637–640. 10.1038/nature02045 14534591

[B26] McGonigle T. P. Miller M. H. Evans D. G. Fairchild G. L. Swan J. A. (1990). A new method that gives an objective measure of colonization of roots by vesicular-arbuscular mycorrhizal fungi. New Phytol. 115, 495–501. 10.1111/j.1469-8137.1990.tb00476.x 33874272

[B27] Messinese E. Mun J. H. Yeun L. H. Jayaraman D. Rouge P. Barre A.. (2007). A novel nuclear protein interacts with the symbiotic DMI3 calcium- and calmodulin-dependent protein kinase of *Medicago truncatula*. Mol. Plant Microbe Interact. 20, 912–921. 10.1094/MPMI-20-8-0912 17722695

[B28] Middleton P. H. Jakab J. Penmetsa R. V. Starker C. G. Doll J. Kalo P.. (2007). An *ERF* transcription factor in *Medicago truncatula* that is essential for Nod factor signal transduction. Plant Cell 19, 1221–1234. 10.1105/tpc.106.048264 17449807 PMC1913751

[B29] Mitra R. M. Gleason C. A. Edwards A. Hadfield J. Downie J. A. Oldroyd G. E.. (2004). A Ca^2+^/calmodulin-dependent protein kinase required for symbiotic nodule development: gene identification by transcript-based cloning. Proc. Natl. Acad. Sci. U.S.A. 101, 4701–4705. 10.1073/pnas.0400595101 15070781 PMC384810

[B30] Oldroyd G. E. (2013). Speak, friend, and enter: signalling systems that promote beneficial symbiotic associations in plants. Nat. Rev. Microbiol. 11, 252–263. 10.1038/nrmicro2990 23493145

[B31] Ovchinnikova E. Journet E. P. Chabaud M. Cosson V. Ratet P. Duc G.. (2011). IPD3 controls the formation of nitrogen-fixing symbiosomes in pea and *Medicago* Spp. Mol. Plant Microbe Interact. 24, 1333–1344. 10.1094/MPMI-01-11-0013 21787150

[B32] Parniske M. (2008). Arbuscular mycorrhiza: the mother of plant root endosymbioses. Nat. Rev. Microbiol. 6, 763–775. 10.1038/nrmicro1987 18794914

[B33] Pimprikar P. Carbonnel S. Paries M. Katzer K. Klingl V. Bohmer M. J. (2016). A CCaMK-CYCLOPS-DELLA complex activates transcription of *RAM1* to regulate arbuscule branching. Curr. Biol. 26, 987–998. 10.1016/j.cub.2016.04.021 27020747

[B34] Radutoiu S. Madsen L. H. Madsen E. B. Felle H. H. Umehara Y. Gronlund M.. (2003). Plant recognition of symbiotic bacteria requires two LysM receptor-like kinases. Nature 425, 585–592. 10.1038/nature02039 14534578

[B35] Singh S. Katzer K. Lambert J. Cerri M. Parniske M. (2014). CYCLOPS, a DNA-binding transcriptional activator, orchestrates symbiotic root nodule development. Cell Host Microbe 15, 139–152. 10.1016/j.chom.2014.01.011 24528861

[B36] Smit P. Raedts J. Portyanko V. Debelle F. Gough C. Bisseling T.. (2005). NSP1 of the GRAS protein family is essential for rhizobial Nod factor-induced transcription. Science 308, 1789–1791. 10.1126/science.1111025 15961669

[B37] Soyano T. Hirakawa H. Sato S. Hayashi M. Kawaguchi M. (2014). Nodule inception creates a long-distance negative feedback loop involved in homeostatic regulation of nodule organ production. Proc. Natl. Acad. Sci. U.S.A. 111, 14607–14612. 10.1073/pnas.1412716111 25246578 PMC4210044

[B38] Tamura K. Peterson D. Peterson N. Stecher G. Nei M. Kumar S. (2011). MEGA5: molecular evolutionary genetics analysis using maximum likelihood, evolutionary distance, and maximum parsimony methods. Mol. Biol. Evol. 28, 2731–2739. 10.1093/molbev/msr121 21546353 PMC3203626

[B39] Tirichine L. Imaizumi-Anraku H. Yoshida S. Murakami Y. Madsen L. H. Miwa H.. (2006). Deregulation of a Ca^2+^/calmodulin-dependent kinase leads to spontaneous nodule development. Nature 441, 1153–1156. 10.1038/nature04862 16810257

[B40] Wang E. Yu N. Bano S. A. Liu C. Miller A. J. Cousins D.. (2014). A H^+^-ATPase that energizes nutrient uptake during mycorrhizal symbioses in rice and *Medicago truncatula*. Plant Cell 26, 1818–1830. 10.1105/tpc.113.120527 24781115 PMC4036588

[B41] Wang W. Shi J. Xie Q. Jiang Y. Yu N. Wang E. (2017). Nutrient exchange and regulation in arbuscular mycorrhizal symbiosis. Mol. Plant 10, 1147–1158. 10.1016/j.molp.2017.07.012 28782719

[B42] Yano K. Yoshida S. Muller J. Singh S. Banba M. Vickers K.. (2008). CYCLOPS, a mediator of symbiotic intracellular accommodation. Proc. Natl. Acad. Sci. U.S.A. 105, 20540–20545. 10.1073/pnas.0806858105 19074278 PMC2629324

[B43] Yoo S. D. Cho Y. H. Sheen J. (2007). *Arabidopsis* mesophyll protoplasts: a versatile cell system for transient gene expression analysis. Nat. Protoc. 2, 1565–1572. 10.1038/nprot.2007.199 17585298

[B44] Zhang X. C. Wu X. Findley S. Wan J. Libault M. Nguyen H. T.. (2007). Molecular evolution of lysin motif-type receptor-like kinases in plants. Plant Physiol. 144, 623–636. 10.1104/pp.107.097097 17449649 PMC1914208

[B45] Zhang X. Dong W. Sun J. Feng F. Deng Y. He Z.. (2015). The receptor kinase CERK1 has dual functions in symbiosis and immunity signalling. Plant J. 81, 258–267. 10.1111/tpj.12723 25399831

