# Supplementary material for: IPD3 and IPD3L Function Redundantly in Rhizobial and Mycorrhizal Symbioses
Source: Front Plant Sci. 2018 Mar 16;9:267. doi: 10.3389/fpls.2018.00267 (PMC5865340; doi:10.3389/fpls.2018.00267)
Supplement: Supplementary file 1 [file DataSheet1.DOCX]

Supplementary Material

**Phosphorylation of IPD3/IPD3L fine-tunes root nodule morphogenesis**

Yue Jin^1^, Zixuan Chen^2,3^, Jirong Huang^1^, Jun Yang^2,3^, Kirankumar S. Mysore^4^, Jiangqi Wen^4^, Nan Yu^1*^, Ertao Wang^2*^

* Correspondence: E.W. (etwang@sibs.ac.cn) and N.Y (nyu@sibs.ac.cn).

# Supplementary Data

*IPD3L* genomic DNAsequence

ATGTCATATGAACTATCTAATTTGTCCAATATGCAAAATATGTCTGTAATATCCGAGGGAAGAATTATGCAAGACGATCCTAAGGCGAATGATTATTCAACTGATTTCAATCAATTTCCTTTAGGGTAATTGTAGTAGGGTTCACAGAACCTGCTATGCCATATCTTTGTTGTGATTTCACTTATAACTAATAACTAGTTCAATGCTTATAATTTGGTTTATTGTCAATTTTTTCCAGAGAACCTGTTGATGAAGTTCAACAATCTAGTAACTTGTTTCTTGCCAAGGTTTGCTTACATCAAACTCTTTGCTGAATCTGAAGCATTGGTTCAACAAGACTTCCATGTTGATTTGATTTCGTTATTAATGCAGGCTTGGTTTCTAAGCGATCAACGAATGACAAGAAGTCGATCTTCTGAATTGAGGTATATACCAATTCATTACACTGTCAATCAATCACAATTGTTGGATCATTTAAAACATTTGATTTCAATCATATATGCCTTAAAAGGGATATAAAGGATGGTTTGTGATTGGTAGAGTATAAAATGTTTTACACTGACGATGCATCAAAATTAATCTCTTATTTATTTGCTTTACTTGTAAGTTAAAAGTTAAAAACTATTCAAAAGAAATATTCAACAACTTATTTGGTACTCTTTATTTGCAGGCAAAGGTATTGTCAGATGCACAATGCTCAAGTAGCACAAGGAATAGAATCCGTTCACATGTTGGCTACTCAGAATGGCAATAACACTCAACAAGAAGTTTCAAATTTAAATGGTTTTAACCACCTTAATCAAAAGGGTGCATTCACATCTCCATCGAATTCGTCTTCGTCAACGTTCAATACACATCAATTGAATGATAACACAGATAAAATCTCTTCGTATGTAAATATGCTGAAAGATACATTAGAACATAAGCGACTTACCAGCCAAATAGCGAAACAAGGCGTGGAAGATAACTCAAATGAACTTTTCAATCCTCAAGAAGATTATTTCCTCCAAACTAGCTTTGATGAAGGGAATGAAAATTGGAACCATCAAAATCCAATATATGTCGAAGGATCCTCTACTATTCAAGTTAAGGATCATGAAGTCATGCAAACACTTGAAGCATCCATAAACCTAATTGACTTGGATGGTTTAGCAAATCAAACAAATCCAATATATTTGAGCTCAGCTTCTCCAAGTGAATCTTCTATTGCTGCAACATTAGTCTCTACTGGTTTCGATGGATGCGACGGTCCATGCATATCAAGCCAAACTCTTTGTGAAAGCTCATGGAATAAAGTTGGAGGAAGTGCAAGTTTGGAAAATAGAGTCAGAGGTATAATATGATTTGATTTGGTTTTATAACAATTTACATTTGCTATTATGAGACGATAATTTTGTTTTTGTGGAACTACAGGTTTCAGAGAACAAAAAATTGATAATCTAAAAGATGATAGAGAGGTATAAAATAATCTTAAATATATATTTAACTAGTTTCTCTAAGGAAGATATTTTGGCTATGTACAATATTTTATAATTTTTGTTTAAATTATGCAGAAGAGAAGTCTAGAAAGATATGCATCTGTAACGTCAGGTATTTCAGGTATTTCAGGTAGCAAGAGTAACTAACTGTTAGTGTTCTTTATAACATATAAAAGAACTTTGATTCTTCTAATTCCATTCCATCACTAACCAGTGAATTAATTATGCAGAGGACAAGGAGAATGCCACAAAAAGACGTAGGGTGGAGCGAGCACGAAAGTATGTAATAATAATAATAGAAAAATTATACTCATCTCTGCTAATGAAAATTTAAAGCCTAATATTTGTACAACTTTATTGTGATGCAGAATGGCAGAGGCAAAGGAAAGGAATTTGATACCATCCATTCCCCCTGATATGCAAGCTGTCTTGAAGCGATGTGAAGACCTTGAGAAGGAAGTTCGATCGTTAAAGCTTAATTTATCCTTCATGAACAGGAAAGATTCTGAACAAACAAAGCAGATAGAGGACCTTCATAAGCAAAATGAGGATTTGACTGATGAAAAAGAACACCTTCTGGAAGAGATTGAAACATTAGTATCAAAAAATGGAAAACTCTAATGTTGTGTTTCGTATTTATTCCTTTCAACTAAGTCAATTACATTCTACATATTCATCTTGTTATACAACAGTCAAGAATTCATTCTCTTATTGACCATGAGAACAATAGTTGGAACCACCAATTGATTTCGTATTATTTTGATAATGTTGTGGCCCACGATATTCTTAAAACTCCTCTGATTAGTCAAGTGGCTGTTAATTTGGAAATTAGAGAAAAATGGTCGTTATTCTGTTAGAAGTGTGTATCGGCTGTGTATGGCAGTCATTGCGGATAATTCATTTTCACATCGCCCTGGTAATTGGGCTAGTATTTGGAGATTGAAGGTCCCTCCGAAAGTGAAAAATTTGTTATGGCGAATTTGTCTTGTCGCGGATGTTTGCCGACGCGTGCAAGGCTGTTGGACAAGGGGGTGAATTGCCCTTCTACGTGTGCTATGTGTGAGGAGAGTTACGAGGATGCGACTCGTGTGTTATTTGATTGTCCGAAAGCGAGAAATGTTTGGTTGAATTGCTCTATAGTAGACAGAGTGAATTCGGTGATGCTTAGCAACAACACAGCAGCTGAAATCACTTCTGTGCTATTACAAGAGTTAACAAAGGAGAAAGCCGAATAGTTTGCTATGAATTTACGGTGGTTATGGAAGAGTCAGAATCTAAGAGTTTGGCAGAACATTTCAGAGATGTGCCAGGCCATTACAGTTCGAGCAAGGCAGCTGTTACATGACTGGAGAGAAGCAAATATCAGAAAATTGTATTCTGATGCAGCTGGAAACATGGCAGAACCGAAGCAAAGTTTGATGCCATCACAAGTTAAGTGGGCAAAACCGCAGCAAGGGAGGTTAAAATGTAATATAGATGGGGCGTTTTCGGAAGCTTTAAACCGTGTTGGTGTTGAGCTATGCAAACGGGATGCTGCTGGAAATTTTAAAAGGCAAAGATGTTGTGGACTAATCCCATATGTACACCGGAGATTGGGGAAGCGTTGGGACTGCTCCATGCAATTCATTGGGTGCATGAACTACAATTTTTAAATGTAGATTTTGAGATGGATGCAAAGAAAGAAAGTTGTAGATTACTTTAATAAGGGCAGCAATGATGTTTCCGAGTTTGGACCAATTTTGGAGCAATATTGGATGAATGTAAAAGATGTCGTAATGGTTATTTCAAAAACACTAAGGTGGAGTTTAGTCGGAGACAAGCGAATGAAGTCGCTCATACTCTTGCTAGAGAAGCCTTACTCTTAA

*IPD3L*cDNA sequence

ATGTCATATGAACTATCTAATTTGTCCAATATGCAAAATATGTCTGTAATATCCGAGGGAAGAATTATGCAAGACGATCCTAAGGCGAATGATTATTCAACTGATTTCAATCAATTTCCTTTAGGAGAACCTGTTGATGAAGTTCAACAATCTAGTAACTTGTTTCTTGCCAAGGCTTGGTTTCTAAGCGATCAACGAATGACAAGAAGTCGATCTTCTGAATTGAGGCAAAGGTATTGTCAGATGCACAATGCTCAAGTAGCACAAGGAATAGAATCCGTTCACATGTTGGCTACTCAGAATGGCAATAACACTCAACAAGAAGTTTCAAATTTAAATGGTTTTAACCACCTTAATCAAAAGGGTGCATTCACATCTCCATCGAATTCGTCTTCGTCAACGTTCAATACACATCAATTGAATGATAACACAGATAAAATCTCTTCGTATGTAAATATGCTGAAAGATACATTAGAACATAAGCGACTTACCAGCCAAATAGCGAAACAAGGCGTGGAAGATAACTCAAATGAACTTTTCAATCCTCAAGAAGATTATTTCCTCCAAACTAGCTTTGATGAAGGGAATGAAAATTGGAACCATCAAAATCCAATATATGTCGAAGGATCCTCTACTATTCAAGTTAAGGATCATGAAGTCATGCAAACACTTGAAGCATCCATAAACCTAATTGACTTGGATGGTTTAGCAAATCAAACAAATCCAATATATTTGAGCTCAGCTTCTCCAAGTGAATCTTCTATTGCTGCAACATTAGTCTCTACTGGTTTCGATGGATGCGACGGTCCATGCATATCAAGCCAAACTCTTTGTGAAAGCTCATGGAATAAAGTTGGAGGAAGTGCAAGTTTGGAAAATAGAGTCAGAGGTTTCAGAGAACAAAAAATTGATAATCTAAAAGATGATAGAGAGAAGAGAAGTCTAGAAAGATATGCATCTGTAACGTCAGGTATTTCAGAGGACAAGGAGAATGCCACAAAAAGACGTAGGGTGGAGCGAGCACGAAAAATGGCAGAGGCAAAGGAAAGGAATTTGATACCATCCATTCCCCCTGATATGCAAGCTGTCTTGAAGCGATGTGAAGACCTTGAGAAGGAAGTTCGATCGTTAAAGCTTAATTTATCCTTCATGAACAGGAAAGATTCTGAACAAACAAAGCAGATAGAGGACCTTCATAAGCAAAATGAGGATTTGACTGATGAAAAAGAACACCTTCTGGAAGAGATTGAAACATTAAA AAATGGTCGTTATTCTGTTAGAAGTGTGTATCGGCTGTGTATGGCAGTCATTGCGGATAATTCATTTTCACATCGCCCTGGTAATTGGGCTAGTATTTGGAGATTGAAGAGTCAGAATCTAAGAGTTTGGCAGAACATTTCAGAGATGTGCCAGGCCATTACAGTTCGAGCAAGGCAGCTGTTACATGACTGGAGAGAAGCAAATATCAGAAAATTGTATTCTGATGCAGCTGGAAACATGGCAGAACCGAAGCAAAGTTTGATGCCATCACAAGTTAAGTGGGCAAAACCGCAGCAAGGGAGGTTAAAATGTAATATAGATGGGGCGTTTTCGGAAGCTTTAAACCGTGTTGGTGTTGAGCTATGCAAACGGGATGCTGCTGGAAATTTTAAAAGGCAAAGATGTTGTGGACTAATCCCATATATTTTGAGATGGATGCAAAGAAAGAAAGTTGTAGATTACTTTAATAAGGGCAGCAATGATGTTTCCGAGTTTGGACCAATTTTGGAGCAATATTGGATGAATGTAAAAGATGTCGTAATGGTTATTTCAAAAACACTAAGGTGGAGTTTAGTCGGAGACAAGCGAATGAAGTCGCT CATACTCTTGCTAGAGAAGCCTTACTCTTAA

**Supplemental Data set S1.**Genomic DNA and cDNA sequence of *IPD3L*. The exons were shaded in grey, and the additional nucleotides of *IPD3L* were shown on a yellow background.

# 2 Supplementary Figures


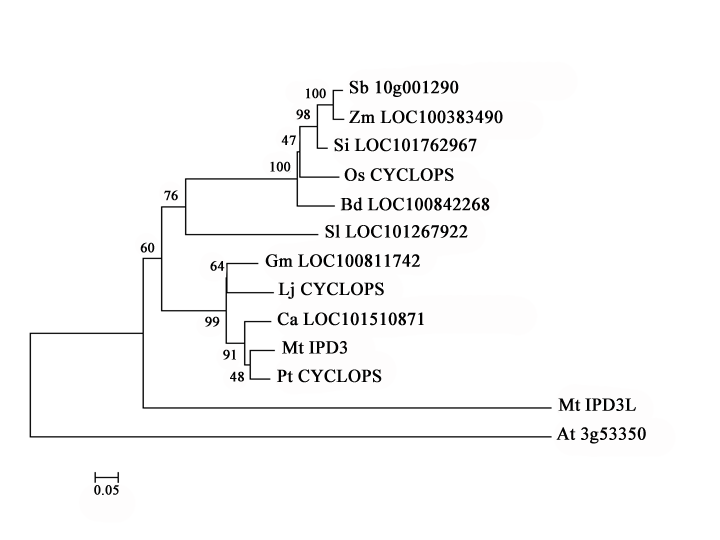


**Figure S1.** Phylogenetic tree of IPD3 and homologous.

The phylogenetic tree of IPD3 from *Glycine max* (Gm), *Arabidopsis thaliana* (At), *Zea mays* (Zm), *Setaria italica* (Si), *Oryza sativa* (Os), *Brachypodium distachyon* (Bd), *Sorghum bicolor* (Sb), *Medicago truncatula* (Mt), *Lotus japonicus* (Lj)*, Cicer arietinum* (Ca), *Solanum lycopersicum* (Sl), *Populus trichocarpa* (Pt)*.* Branch support was obtained from 1000 bootstrap repetitions.


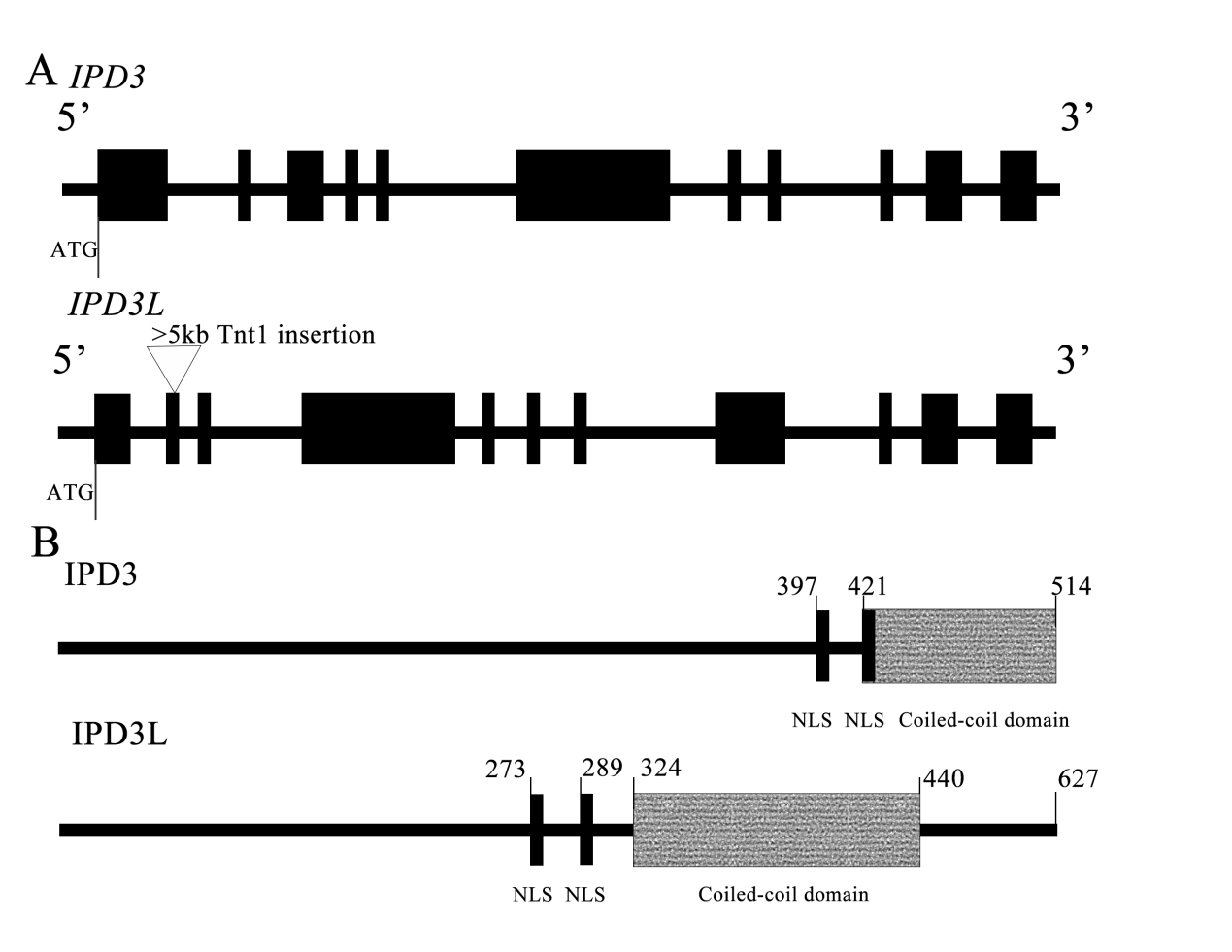


**Figure S2.** Gene and protein structures of *IPD3* and *IPD3L*.

(A) Schematic representation of the exon-intron structure of the *IPD3* and *IPD3L* gene. Identified mutation of *ipd3l* is indicated. (B) Schematic representation of the domain structure of IPD3 and IPD3L proteins. The C-terminal coiled-coil domain, and two predicted nuclear localization sites (NLS) are indicated.


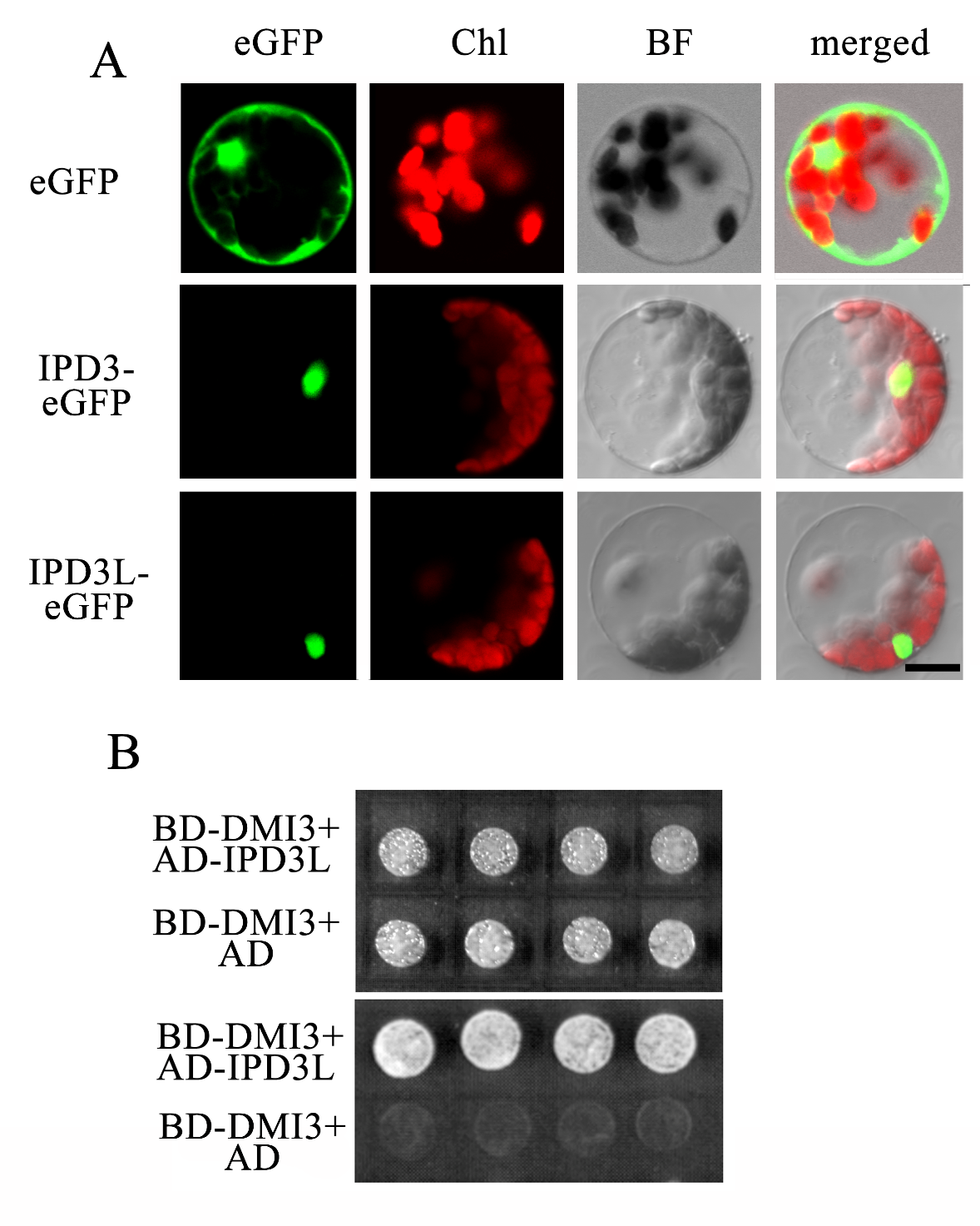


**Figure S3.** IPD3L localized to the nucleus and interacted with DMI3 in yeast.

(A) Expression of IPD3 and IPD3L proteins in *Arabidopsis* mesophyll cells. The green fluorescent protein of IPD3 and IPD3L were observed in the nucleus. All the images were recorded with the scale (scale bar, 15 μm). (B) IPD3L and DMI3-Gal4 DNA binding domain fusions activated the Gal4 system in *S. cerevisiae*. *S. cerevisiae* was grown on media with selection for growth.


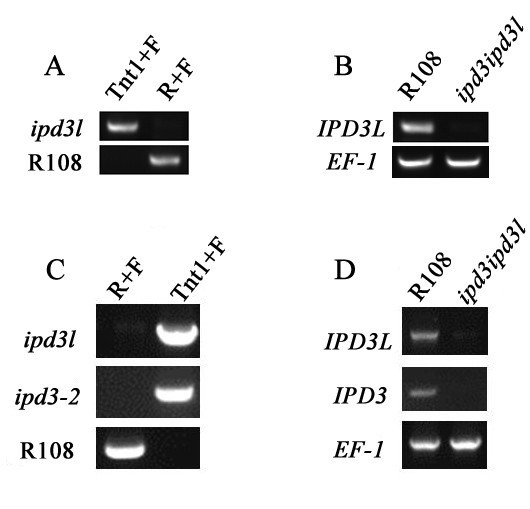


**Figure S4.** Identification of *ipd3l* and *ipd3l ipd3-2* mutants.

(A, C) Genotyping analyses of *IPD3* and *IPD3L* in genomic DNA from wild-type (R108), *ipd3-2* and *ipd3l* in *M. truncatula*. (B, D) PCR analyses of *IPD3* and *IPD3L* transcript levels in total RNA samples from *M. truncatula* roots at 1 wpi with *S. meliloti* 1021.


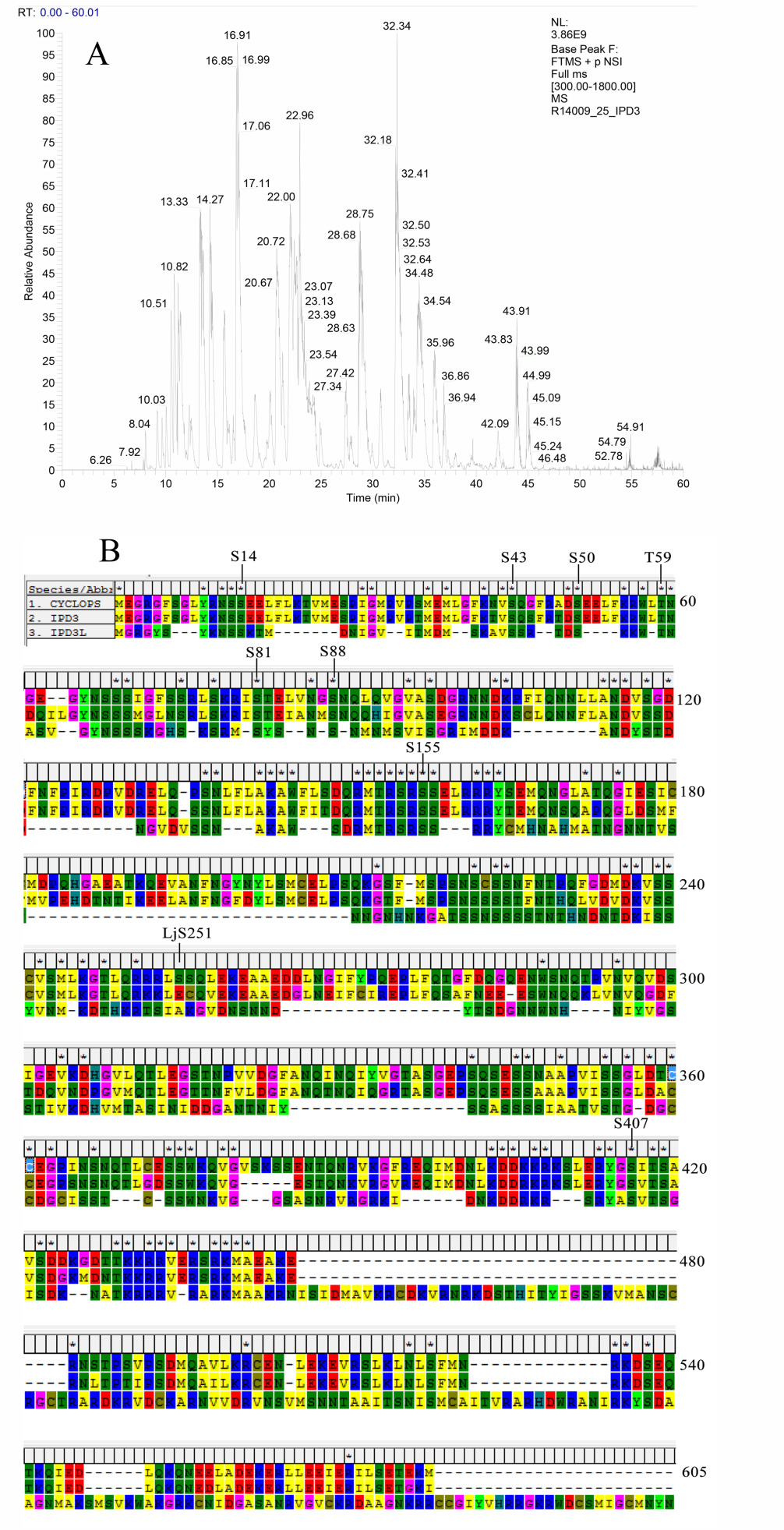


**Figure S5.** Identification of phosphorylated serine residues of IPD3.

(A) MALDI-TOF MS analysis of IPD3. (B) Alignment of CYCLOPS, IPD3 and IPD3L amino acid sequences. The identified phosphorylation sites are conserved in CYCLOPS, IPD3 and IPD3L amino acid sequences, except for T251 of CYCLOPS.


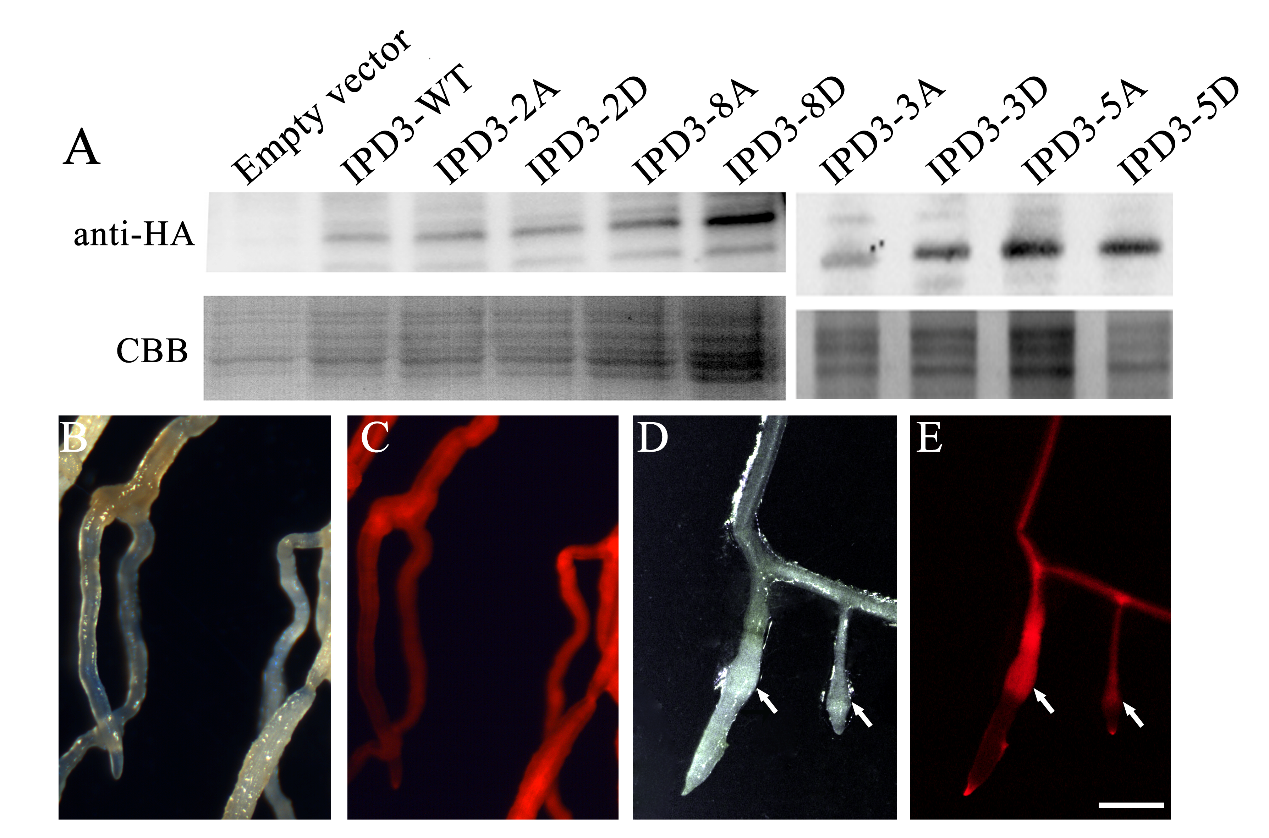


**Figure S6.** IPD3 protein levels in the *ipd3l ipd3-2* hairy roots transformed with phosphoablative/mimetic IPD3 mutant versions and the phenotype of the *ipd3l ipd3-2* hairy roots transformed with *DMI3 1-311*.

(A) Protein blot probed with anti-HA-HRP antibody demonstrating that 3xHA-IPD3-WT and mutant derivatives were expressed at the similar levels in the transformed roots of the *ipd3l ipd3-2* mutant. Protein extracts were prepared from transgenic roots four weeks post inoculation with *sm1021*. Molecular weight of 3xHA-IPD3 is 55 kDa. No protein band equal in size to 3xHA-IPD3 was detected in roots transformed with the empty vector control. Coomassie blue-stained blot shows equal sample loading of the gel. (B, C) The normal root of *ipd3l ipd3-2* hairy root transforming DMI3 1-311. (D, E) The surface protuberances of *ipd3l ipd3-2* hairy root transforming DMI3 1-311. All the images were recorded with the scale (scale bar, 1 mm).


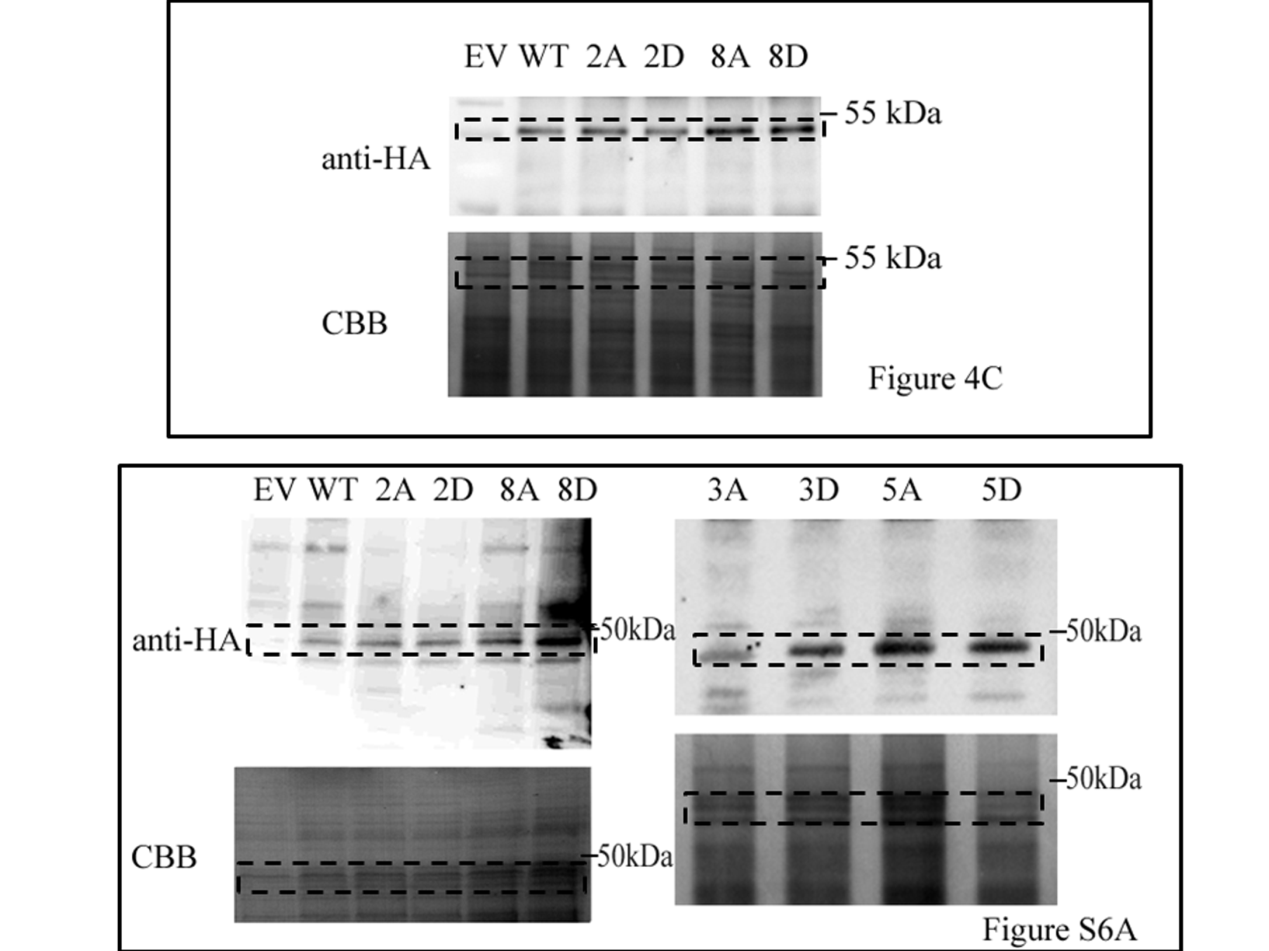


**Figure S7**. Uncropped images of immunoblotting results in Figure 4C and Figure S6A.

# 3 Supplementary Tables

**Table S1.** Primers/plasmids used in this study.

| primers used for genotyping |  |
| --- | --- |
| NF14178-F | TGTCATATGAACTATCTAATTTGTCCAAT |
| NF14178-R | TAATGTTTCAATCTCTTCCAGAAG |
| NF5939-F | TTAAAGTGTTGAAATGGAAGGGAG |
| NF5939-R | TTGTGAAAGACCACATCATTTACCT |
| Tnt1-R1 | TGTAGCACCGAGATACGGTAATTAACAAGA |
|  |  |
| primers used for point mutation |  |
| IPD3-3HA-F | TTGGATCCATGTACCCATACGATGTTCCAGATTACGCTTACCCATACGATGTTCCAGATTACGCTTACCCATACGATGTTCCAGATTACGCTATGGAAGGGAGAGGATTTTCTGG |
| IPD3-3HA-R | TTGAGCTCTCAAATCTTTCCAGTTTCTGATAGAAT |
| IPD3-S14A-F | TTATACAAGAATTCAGCTGAGGAGTTATTCTTG |
| IPD3-S14A-R | CAAGAATAACTCCTCAGCTGAATTCTTGTATAA |
| IPD3-S14D-F | TTATACAAGAATTCAGATGAGGAGTTATTCTTG |
| IPD3-S14D-R | CAAGAATAACTCCTCATCTGAATTCTTGTATAA |
| IPD3-S43A-F | GGATTCAAGACTGTTGCACAAAGCTTTCGCACC |
| IPD3-S43A-R | GGTGCGAAAGCTTTGTGCAACAGTCTTGAATCC |
| IPD3-S43D-F | GGATTCAAGACTGTTGATCAAAGCTTTCGCACC |
| IPD3-S43D-R | GGTGCGAAAGCTTTGATCAACAGTCTTGAATCC |
| IPD3-S50A-F： | AAGCTTTCGCACCGATGCTGAAGAGCTTTTCAAAC |
| IPD3-S50A-R： | GTTTGAAAAGCTCTTCAGCATCGGTGCGAAAGCTT |
| IPD3-S50D-F： | AAGCTTTCGCACCGATGATGAAGAGCTTTTCAAAC |
| IPD3-S50D-R： | GTTTGAAAAGCTCTTCATCATCGGTGCGAAAGCTT |
| IPD3-T59A-F | TTCAAACGCTGGCTAGCAAATGATCAAGAGGG |
| IPD3-T59A-R | CCCTCTTGATCATTTGCTAGCCAGCGTTTGAA |
| IPD3-T59D-F | TTCAAACGCTGGCTAGATAATGATCAAGAGGG |
| IPD3-T59D-R | CCCTCTTGATCATTATCTAGCCAGCGTTTGAA |
| IPD3-S81A-F | TTGTCGAAGAGAATAGCAACTGAAATAGCTAAT |
| IPD3-S81A-R | ATTAGCTATTTCAGTTGCTATTCTCTTCGACAA |
| IPD3-S81D-F | TTGTCGAAGAGAATAGATACTGAAATAGCTAAT |
| IPD3-S81D-R | ATTAGCTATTTCAGTATCTATTCTCTTCGACAA |
| IPD3-S88A-F | GAAATAGCTAATATGGCTAATCAACAACACATT |
| IPD3-S88A-R | AATGTGTTGTTGATTAGCCATATTAGCTATTTC |
| IPD3-S88D-F | GAAATAGCTAATATGGATAATCAACAACACATT |
| IPD3-S88D-R | AATGTGTTGTTGATTATCCATATTAGCTATTTC |
| IPD3-S155A-F： | TACAAGAAGCCGGGCATCTGAATTGCGGGCG |
| IPD3-S155A-R： | CGCCCGCAATTCAGATGCCCGGCTTCTTGTA |
| IPD3-S155D-F： | TACAAGAAGCCGGGATTCTGAATTGCGGGCG |
| IPD3-S155D-R： | CGCCCGCAATTCAGAATCCCGGCTTCTTGTA |
| IPD3-S407A-F | CTAGAAAGATATGGAGCTGTAACATCAGCTGTT |
| IPD3-S407A-R | AACAGCTGATGTTACAGCTCCATATCTTTCTAG |
| IPD3-S407D-F | CTAGAAAGATATGGAGATGTAACATCAGCTGTT |
| IPD3-S407D-R | AACAGCTGATGTTACATCTCCATATCTTTCTAG |
|  |  |
| primers used in real-time PCR |  |
| IPD3-QPCR-F | GCGCTCAAGAAAAATGGCTGAAGC |
| IPD3-QPCR-R | GCTTTAGTGATCGAACTTCCTTCTCAAGG |
| IPD3L-qPCR-F1 | TGGCTACTCAGAATGGCAATAACAC |
| IPD3L-qPCR-R1 | CTGGTAAGTCGCTTATGTTCTAATGTATCT |
| NIN-qF | GCAATGTGGGGATTTAGAGATT |
| NIN-qR | GGAAGATTGAGAGGGGAAG |
| ERN1-qF | GGAAGATGGTGCTGTTGCTT |
| ERN1-qR | TGTTGGATTGTGAACCTGACTC |
| FLOT4-qF | CTGAGGCAGTACGCGGACTTG |
| FLOT4-qR | AGGTGGTAACATTCCCGTTTG |
| RIP-qF | TAGGGAAAAACGCATTGGAG |
| RIP-qR | ACAACAGGGCCTTTGCATAC |
| Vapyrin -qF | TCATCCTCCACAACAACAAGGT |
| Vapyrin -qR | TCAAGCACTTCTCTTATGTCATCCATTG |
| MtENDO11-qF | TTCTTGTACTCGCTAGGGTTAGTGTT |
| MtENDO11-qR | GAGGCTTGTAAGTAGGAGGAGGC |
| ENOD40-qF | CAATCACTCTATCTATGTAGCACTG |
| ENOD40-qR | CTCAAAGGAAGACAACACCATC |
|  |  |
| Primers used in over-expression transformation |  |
| IPD3p- SacI-F1 | AAGAGCTCAAGTGGAGTCAAAAGAAATAGTTTATATG |
| IPD3p-SpeI-R1 | TTACTAGTGAAACACTTGAATGATGCTTGAACTTATT |
| IPD3Lp-BamHI-F | TTGGATCCAACTACCTAAAGATAAGTTTCTGCTTTTC |
| IPD3Lp-SacI (also in pEntry) | TTGAGCTCTCTGCAGTTCAACAAAATCAAAAG |
| IPD3L-SalI-F (also in pEntry) | AAAGTCGACAATGTCATATGAACTATCTAATTTGTCC |
| IPD3L-NotI-R | AAAGCGGCCGCTTAAGAGTAAGGCTTCTCTAGCAA |
| IPD3-F (also in pEntry) | CACCATGGAAGGGAGAGGATTTTCTG |
| IPD3-R (also in pEntry) | TCAAATCTTTCCAGTTTCTGATAGAA |
| DMI3 1-311-BamHI-F | AAGGATCCATGGGATATGGAACAAGAAAACTC |
| DMI3 1-311-SacI-R | AAGAGCTCTCAGGCTTTCTCACCTTTGACC |
|  |  |
| primers used in expression *in vitro* |  |
| CCaMK-XbaI-F | AATCTAGAATGGGATATGGAACAAGAAAACTC |
| CCaMK-PstI-R | AAACTGCAGTTATGGACGAATAGAAGAGAGAACTAC |
| IPD3-BamHI-F | TTGGATCCATGGAAGGGAGAGGATTTTCTG |
| IPD3-SalI-R | TTTGTCGACTCAAATCTTTCCAGTTTCTGATAGAAT |
| plasmids used in this study |  |
| Entry clones |  |
| pENTR:IPD3 | Phusion PCR product of the 1542 bp *IPD3* nucleotide sequence amplified from mRNA of *M. trunctula* with IPD3 fwd/rev and cloned into pENTR/D-TOPO |
| pENTR:3xHA-IPD3 | Phusion PCR product of the 1626 bp *IPD3* nucleotide (containing 3xHA in primers) sequence amplified from pENTR:IPD3 with IPD3-3HA-F/R cloned into pENTR/D-TOPO |
| pENTR:3xHAIPD3S50A | Site directed mutagenesis Phusion PCR with *IPD3-*S50A CDS + coding sequence_fwd/rev on pENTR:3xHA-IPD3 |
| pENTR:3xHAIPD3T59A | Site directed mutagenesis Phusion PCR with *IPD3-*T59A CDS + coding sequence_fwd/rev on pENTR:3xHA-IPD3 |
| pENTR:3xHAIPD3S88A | Site directed mutagenesis Phusion PCR with *IPD3-*S88A CDS + coding sequence_fwd/rev on pENTR:3xHA-IPD3 |
| pENTR:3xHAIPD3S81A | Site directed mutagenesis Phusion PCR with *IPD3-*S81A CDS + coding sequence_fwd/rev on pENTR:3xHA-IPD3 |
| pENTR:3xHAIPD3S50A-S155A | Site directed mutagenesis Phusion PCR with *IPD3*-S155A CDS + coding sequence_fwd/rev on pENTR:3xHA-IPD3-S50A |
| pENTR:3xHAIPD3S43A-S50A-S155A | Site directed mutagenesis Phusion PCR with *IPD3*-S43A CDS + coding sequence_fwd/rev on pENTR:3xHA-IPD3 S50A-S155A |
| pENTR:3xHAIPD3S14A-S43A-S50A-S155A | Site directed mutagenesis Phusion PCR with *IPD3*-S14A CDS + coding sequence_fwd/rev on pENTR:3xHA-IPD3S43A-S50A-S155A |
| pENTR:3xHAIPD3S14A-S43A-S50A-S155A-S407A | Site directed mutagenesis Phusion PCR with *IPD3*-S407A CDS + coding sequence_fwd/rev on pENTR:3xHA-IPD3S43A-S50A-S155A |
| pENTR:3xHAIPD3S14A-S43A-S50A-T59A-S155A-S407A | Site directed mutagenesis Phusion PCR with *IPD3*-T59A CDS + coding sequence_fwd/rev on pENTR:3xHA-IPD3S43A-S50A-S155A-S407A |
| pENTR:3xHAIPD3S14A-S43A-S50A-T59A-S81A-S155A-S407A | Site directed mutagenesis Phusion PCR with *IPD3*-S81A CDS + coding sequence_fwd/rev on pENTR:3xHA-IPD3S43A-S50A-T59A-S155A-S407A |
| pENTR:3xHAIPD3S14A-S43A-S50A-T59A-S81A-S88A-S155A-S407A | Site directed mutagenesis Phusion PCR with *IPD3*-S88A CDS + coding sequence_fwd/rev on pENTR:3xHA-IPD3S43A-S50A-T59A-S81A-S155A-S407A |
| pENTR:3xHAIPD3S50D | Site directed mutagenesis Phusion PCR with *IPD3*-S50D CDS + coding sequence_fwd/rev on pENTR:3xHA-IPD3 |
| pENTR:3xHAIPD3S50D-155D | Site directed mutagenesis Phusion PCR with *IPD3*-S155D CDS + coding sequence_fwd/rev on pENTR:3xHA-IPD3-S50D |
| pENTR:3xHAIPD3S43D-S50D-S155D | Site directed mutagenesis Phusion PCR with *IPD3*-S43D CDS + coding sequence_fwd/rev on pENTR:3xHA-IPD3 S50D-S155D |
| pENTR:3xHAIPD3S14D-S43D-S50D-S155D | Site directed mutagenesis Phusion PCR with *IPD3*-S14A CDS + coding sequence_fwd/rev on pENTR:3xHA-IPD3S43D-S50D-S155D |
| pENTR:3xHAIPD3S14D-S43D-S50D-S155D-S407D | Site directed mutagenesis Phusion PCR with *IPD3-*S407D CDS + coding sequence_fwd/rev on pENTR:3xHA-IPD3S43D-S50D-S155D |
| pENTR:3xHAIPD3S14D-S43D-S50D-T59D-S155D-S407D | Site directed mutagenesis Phusion PCR with *IPD3*-T59D CDS + coding sequence_fwd/rev on pENTR:3xHA-IPD3S43D-S50D-S155D-S407D |
| pENTR:3xHAIPD3S14D-S43D-S50D-T59D-S81D-S155D-S407D | Site directed mutagenesis Phusion PCR with *IPD3*-S81D CDS + coding sequence_fwd/rev on pENTR:3xHA-IPD3S43D-S50D-T59D-S155D-S407D |
| pENTR:3xHAIPD3S14D-S43D-S50D-T59D-S81D-S88D-S155D-S407D | Site directed mutagenesis Phusion PCR with *IPD3*-S88D CDS + coding sequence_fwd/rev on pENTR:3xHA-IPD3S43D-S50D-T59D-S81D-S155D-S407D |
| pENTR:IPD3L | Phusion PCR product of the 1890 bp *IPD3* nucleotide sequence amplified from mRNA of *M. trunctula* with IPD3L fwd/rev and cloned into pENTR/D-TOPO |
| pENTR:DMI3 | Phusion PCR product of the 1572 bp *IPD3* nucleotide sequence amplified from mRNA of *M. trunctula* with DMI3 fwd/rev and cloned into pENTR/D-TOPO |
| pENTR:DMI3 1-311 | Phusion PCR product of the 933 bp *IPD3* nucleotide sequence amplified from mRNA of *M. trunctula* with DMI3 fwd/rev and cloned into pENTR/D-TOPO |
| pENTR:IPD3L promoter | Phusion PCR product of the 3000 bp *IPD3L* promoter nucleotide sequence amplified from genomic DNA of *M. trunctula* with IPD3Lp fwd/rev and cloned into pENTR/D-TOPO |
| pENTR:IPD3 promoter | Phusion PCR product of the 1048 bp *IPD3* promoter nucleotide sequence amplified from genomic DNA of *M. trunctula* with IPD3p fwd/rev and cloned into pENTR/D-TOPO |
| Plasmids for subcellular localization, Y2H and protein expression analysis |  |
| IPD3L-eGFP | Phusion PCR product of *IPD3L*Δstop amplified from pENTR:IPD3L with *IPD3L*Δstop_fwd/rev and cloned into pSAT4-eGFP-N1 with HindIII and BamHI；pSAT4-eGFP-N1 was from pSAT4-nEYFP-N1 by replace nEYFP using eGFP |
| IPD3-eGFP | Phusion PCR product of *IPD3*Δstop amplified from pENTR:IPD3 with *IPD3*Δstop_fwd/rev and cloned into pSAT4-eGFP-N1 with SalI and BamHI |
| AD-IPD3L | LR reaction (Invitrogen) of pENTR:IPD3L and Gateway modified pGADT7-GW |
| AD-IPD3 | LR reaction (Invitrogen) of pENTR:IPD3 and Gateway modified pGADT7-GW |
| BD-DMI3 | LR reaction (Invitrogen) of pENTR:DMI3 and Gateway modified pGBKT7-GW |
| pIPD3:GUS | LR reaction (Invitrogen) of pENTR:IPD3 promoter and Gateway vector pBGWFS7 |
| pIPD3L:GUS | LR reaction (Invitrogen) of pENTR:IPD3L promoter and Gateway vector pBGWFS7 |
| 6XHIS-IPD3 | The full-length IPD3 CDS was cloned into pET 28a with BamHI and SalI |
| MBP-DMI3 | The full-length MID3 CDS was cloned into pMAL-C2X with XbaI and PstI |
| Plasmids for *M. trunctula* hairy root transformation |  |
| pK7WG2-R (*LjUBQp*) | The p35S promoter was replaced by *LjUBQ* promoter in pK7WG2-R with appropriate restriction enzyme cleavage sites |
| IPD3p:IPD3L | The p35S promoter was replaced by 1.1K IPD3 promoter with appropriate restriction enzyme cleavage sites and then cloned to the modified vector pK7WG2-R by LR reaction |
| pUB:3xHA-IPD3 or phosphosite mutants | LR reaction (Invitrogen) of pENTR:3xHA-IPD3 or phosphosite mutants (listed in Entry clones) and pK7WG2-R(*LjUBQp*) |

**Table S2.** Root nodule phenotype in *ipd3l ipd3-2* transformed with different phosphoablative *IPD3* mutations.

| Plant Genotype | Transgene | Pink | White | Plant |
| --- | --- | --- | --- | --- |
| R108 | *LjUBQ* | 10.5±0.5 | 2±0 | 5/5 |
| *ipd3l ipd3-2* | *LjUBQ: 3XHA-IPD3-T59A* | 3.4±0.87 | 3.05±1.36 | 8/8 |
| *ipd3l ipd3-2* | *LjUBQ: 3XHA-IPD3-S81A* | 3.5±1.43 | 1±0.52 | 11/11 |
| *ipd3l ipd3-2* | *LjUBQ: 3XHA-IPD3-S88A* | 4.2±1.46 | 0.8±0.8 | 10/10 |

These plants were inoculated with *S. meliloti 1021* and analyzed at 4 wpi. White nodule refers to the small pale nodules containing no or very few bacteroids at 4 wpi.

**Table S3.** Restoration analysis of root symbiosis on *ipd3l ipd3-2* hairy roots by phosphoablative/mimetic IPD3 mutant versions.

| Plant Genotype | Transgene | Pink* | White* | Plant^#^ |
| --- | --- | --- | --- | --- |
| *ipd3l ipd3-2* | *LjUBQ* | 0 | 0 | 0/13 |
| *ipd3l ipd3-2* | *LjUBQ:3XHA-IPD3* | 6.23±0.87 | 11±2.41 | 19/19 |
| *ipd3l ipd3-2* | *LjUBQ:3XHA-IPD3-S50A-S155A* | 0 | 0 | 0/16 |
| *ipd3l ipd3-2* | *LjUBQ:3XHA-IPD3-S43A-S50A-S155A* (*IPD3-3A*) | 0 | 0 | 0/30 |
| *ipd3l ipd3-2* | *LjUBQ:3XHA-IPD3-S14A-S43A-S50A-S155A-S407A* (*IPD3-5A*) | 0 | 0 | 0/28 |
| *ipd3l ipd3-2* | *LjUBQ:3HA-IPD3-S14A-S43A-S50A-S59A-S81A-S88A-S155A-407A* (*IPD3-8A*) | 0 | 0 | 0/28 |
| *ipd3l ipd3-2* | *LjUBQ:3XHA-IPD3-S50D-S155D* (*IPD3-2D*) | 4.33±0.45^ | 3.55±0.70^ | 38/40 |
| *ipd3l ipd3-2* | *LjUBQ:3XHA-IPD3-S43D-S50D-S155D* (*IPD3-3D*) | 4.7±2.77^ | 12.47±9.20 | 25/25 |
| *ipd3l ipd3-2* | *LjUBQ:3XHA-IPD3-S14D-S43D-S50D-S155D-S407D* (*IPD3-5D*) | 3.2±1.46^ | 7.1±6.46 | 16/17 |
| *ipd3l ipd3-2* | *LjUBQ:3XHA-IPD3-S14D-S43D-S50D-S59D-S81D-S88D-S155D-407D* (*IPD3-8D*) | 0.53±0.31^ | 1.35±0.51^ | 8/17 |

These plants were inoculated with *S. meliloti 1021* and analyzed at 4 wpi. White nodule refers to the small pale nodules containing no or very few bacteroids at 4 wpi.

*Average number of nodules per nodulated root system ± standard deviation.

^#^ Number of root systems with nodules per number of total root systems analyzed.

^ indicates a significant decrease compared to the control (*LjUBQ:3XHA-IPD3*) with Student’s t-test (*P≤0.05).

**Table S4.** Analysis of spontaneous nodules triggered on the *ipd3l ipd3-2* hairy roots by phosphoablative/mimetic IPD3 mutant versions.

| Plant Genotype | Transgene | TypeI-spn* | TypeII-spn* | TypeIII-spn* | Plant^#^ |
| --- | --- | --- | --- | --- | --- |
| *ipd3l ipd3-2* | *LjUBQ* | 0 | 0 | 0 | 0/9 |
| *ipd3l ipd3-2* | *LjUBQ: 3XHA-IPD3* | 0 | 0 | 0 | 0/7 |
| *ipd3l ipd3-2* | *LjUBQ: 3XHA-IPD3-S50A-S155A* | 0 | 0 | 0 | 0/11 |
| *ipd3l ipd3-2* | *LjUBQ:3XHA-IPD3-S14A-S43A-S50A-T59A-S81A-S88A-S155A-407A* (*IPD3-8A*) | 0 | 0 | 0 | 0/7 |
| *ipd3l ipd3-2* | *LjUBQ:3XHA-IPD3-S50D-S155D* (*IPD3-2D*) | 8±2.83 | 0 | 0 | 6/10 |
| *ipd3l ipd3-2* | *LjUBQ:3XHA-IPD3-S14D-S43D-S50D-T59D-S81D-S88D-S155D-407D* (*IPD3-8D*) | 2.4±1.88^^1^ | 0 | 0 | 8/14 |
| *dmi3-1* | *LjUBQ: DMI3 1-311* | 4.22±1.61 | 0 | 0 | 9/16 |
| *dmi3-1* | *LjUBQ* | 0 | 0 | 0 | 0/10 |
| *dmi3-1* | *LjUBQ: 3XHA-IPD3* | 0 | 0 | 0 | 0/19 |
| *dmi3-1* | *LjUBQ: 3XHA-IPD3-S50A-S155A* | 0 | 0 | 0 | 0/8 |
| *dmi3-1* | *LjUBQ:3XHA-IPD3-S50D-S155D* (*IPD3-2D*) | 0 | 2.33±0.66^^2^ | 0 | 6/18 |
| *dmi3-1* | *LjUBQ:3XHA-IPD3-S14D-S43D-S50D-T59D-S81D-S88D-S155D-407D* (*8D*) | 0 | 0 | 0.75±0.25^^2^ | 4/14 |
| *dmi2-1* | *LjUBQ: 3XHA-IPD3* | 0 | 0 | 0 | 0/23 |
| *dmi2-1* | *LjUBQ: 3XHA-IPD3-S50D-S155D* | 0 | 2.75±0.86 | 0.25±0.25 | 4/21 |

These plants were analyzed at 8 weeks after transformation without *Sm 1021*.

*Average number of spontaneous nodules per spontaneously nodulated root system ± standard deviation. Different nodules types correspond to those in **Figure 6A**.

**^#^** Number of root systems with spontaneous nodules per number of total root systems analyzed. Spontaneous nodules were scored at 8 weeks post transformation.

^^1^ indicates a significant decrease compared to the control (*LjUBQ:3XHA-IPD3-2D*) with Student’s t-test (*P≤0.05). ^^2^ indicates a significant decrease compared to the control (*LjUBQ:DMI3 1-311*) with Student’s t-test (*P≤0.05).

**Table S5.** Root nodule phenotype in *dmi2-1* and *dmi3-1* transformed with phosphoablative/mimetic *IPD3* mutations.

| Plant Genotype | Transgene | Pink | White | Plant |
| --- | --- | --- | --- | --- |
| A17 | *LjUBQ* | 9.87±1.47 | 3.46±2.13 | 16/16 |
| *dmi3-1* | *LjUBQ* | 0 | 0 | 0/18 |
| *dmi3-1* | *LjUBQ:* *3XHA-IPD3-S50A-S155A* | 0 | 0 | 0/21 |
| *dmi3-1* | *LjUBQ:3XHA-IPD3-S14A-S43A-S50A-T59A-S81A-S88A-S155A-407A* | 0 | 0 | 0/24 |
| *dmi3-1* | *LjUBQ:* *3XHA-IPD3-S50D-S155D* | 0 | 0 | 0/28 |
| *dmi3-1* | *LjUBQ:3XHA-IPD3-S14D-S43D-S50D-T59D-S81D-S88D-S155D-407D* | 0 | 0 | 0/20 |
| *dmi2-1* | *LjUBQ:* *3XHA-IPD3-S50D-S155D* | 0 | 0 | 0/13 |

These plants were inoculated with *S. meliloti 1021* and analyzed at 4 wpi.

White nodule refers to the small pale nodules containing no or very few bacteroids at 4 wpi.
